# The Mechanism Underlying the Abnormal Expression of α‐Synuclein in the Cortical Lesions of Patients With FCD Type IIb and TSC

**DOI:** 10.1002/cns.70893

**Published:** 2026-04-24

**Authors:** Li Zhang, Jian‐Ping Song, Jun Huang, Ping Liang, Hui Yang, Chun‐Qing Zhang, Kai‐Feng Shen

**Affiliations:** ^1^ Department of Neurosurgery Epilepsy Research Center of PLA, Xinqiao Hospital, Army Medical University Chongqing China; ^2^ Chongqing Institute for Brain and Intelligence, Guang Yang Bay Laboratory Chongqing China; ^3^ Department of Neurosurgery Children's Hospital of Chongqing Medical University, National Clinical Research Center for Child Health and Disorders, Ministry of Education Key Laboratory of Child Development and Disorders, Chongqing Key Laboratory of Pediatrics Chongqing China

**Keywords:** FCD IIb, GLT‐1, mTOR, TSC, α‐Syn

## Abstract

**Aims:**

This study aims to investigate the underlying mechanisms of abnormal expression of α‐synuclein (α‐syn) in cortical lesions of focal cortical dysplasia IIb (FCD IIb) and tuberous sclerosis complex (TSC).

**Methods:**

Cortical lesions of patients with FCD IIb and TSC were obtained during the surgery, FCD rats were generated by in utero X‐ray radiation. Immunostaining, RT–PCR, Western blotting, and electroencephalography recording were conducted in this study. α‐syn was intraventricularly injected; mTOR inhibitor (rapamycin) and glutamate transporter‐1 (GLT‐1) enhancer (ceftriaxone sodium) were intraperitoneally injected before the following experiments.

**Results:**

mTOR and p‐mTOR expressed in the dysmorphic neurons, vimentin‐positive balloon cells, and giant cells, whereas the mTOR/p‐mTOR ratio was decreased in FCD IIb and TSC lesions. α‐syn was decreased, while p‐α‐syn was increased in the cortex of FCD animals, whereas they were rescued by inhibition of mTOR with rapamycin. The expression of GLT‐1 was decreased and modulated by rapamycin in FCD animals. Enhancement of the function of GLT‐1 with ceftriaxone ameliorated α‐synucleinopathy and seizure activities in FCD animals. Additionally, intracerebroventricular injection of α‐syn exerted anti‐seizure effects.

**Conclusions:**

Our results showed that modulation of the mTOR/GLT‐1 pathway ameliorated α‐synucleinopathy and seizure activities in FCD, providing insights for understanding the epileptogenic mechanisms of FCD IIb and TSC.

## Graphical abstract

The mTOR/GLT‐1 pathway abnormality induces α‐synucleinopathy, contributing to seizures in FCD lesions, which were rescued by treatment with the mTOR inhibitor Rapa or the GLT‐1 enhancer Cef. Intracerebroventricular α‐syn administration attenuated PTZ‐induced seizures in FCD models.

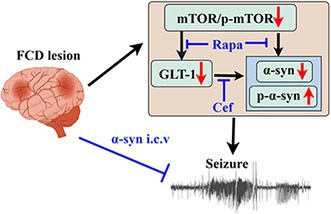

## 1 Introduction

Malformations of cortical development (MCD) encompasses a spectrum of neurodevelopmental disorders that are a common cause of neurodevelopmental delay and pediatric epilepsy [1]. Focal cortical dysplasia IIb (FCD IIb) and tuberous sclerosis complex (TSC) represent two cardinal MCD subtypes [2]. FCD and TSC demonstrate convergent pathological characteristics, marked by cortical dyslamination, together with the presence of dysmorphic neurons (DNs), balloon‐like cells (BCs), and giant cells (GCs) [3]. Children with FCD IIb and TSC commonly develop seizures in infancy or toddlerhood, progressing to drug‐resistant epilepsy ultimately [4]. Studies have indicated that alterations in genetically linked neurodevelopmental‐ and neuroinflammatory‐associated molecules play substantial roles in the pathogenesis of FCD IIb and TSC, with DNs serving as ictogenic sources for epilepsy [5, 6, 7, 8]. Nevertheless, the precise mechanisms linking FCD IIb and TSC remain incompletely characterized. Further investigation may reveal foundational insights into pediatric drug‐resistant epilepsy pathogenesis.

α‐Synuclein (α‐syn), a presynaptic protein in the synuclein family [9], physiologically regulates synaptic vesicle trafficking and plasticity maintenance [10]. Pathogenic phosphorylation at Ser129 triggers cytoplasmic accumulation of pSer129‐α‐syn, forming neuronal inclusion bodies that characterize synucleinopathies in neurodegenerative disorders [11]. However, the precise function of p‐α‐syn has yet to be fully understood. Our previous study demonstrated dysregulated α‐syn proteostasis in FCD IIb and TSC cortical lesions, with increased interactions with NMDAR2A and NMDAR2B [12]. However, the epileptogenic mechanisms mediated by α‐syn and the mechanisms driving its dysregulation in cortical lesions in patients with FCD IIb and TSC await elucidation.

The classical mTOR signaling pathway critically regulates cellular proteostasis, growth, and synaptogenesis [13, 14]. and mTOR hyperactivation is pathognomonic in FCD lesions [15]. Crucially, mTOR modulated α‐syn transcription and translation in Parkinson's disease models [16], while rapamycin‐mediated inhibition of mTOR reduced p‐α‐syn burden in neuronal cultures [17], suggesting mTOR as a master regulator of α‐syn pathophysiology. GLT‐1, the predominant astrocytic glutamate transporter, localizes to synaptic compartments [18] and critically regulates extracellular glutamate clearance [19]. Its dysregulation causes glutamate excitotoxicity via NMDA receptor hyperactivation, driving epileptogenesis [20]. Crucially, GLT‐1 influenced α‐syn expression and function in Parkinson's models [21, 22], while GLT‐1 deficiency disrupts synaptic transmission and accelerates α‐syn progression [22]. Notably, the expression of GLT‐1 was directly suppressed by mTOR in a pilocarpine‐induced status epileptic model [23]. Given the concurrent dysregulation of mTOR signaling and GLT‐1 expression in neurological disorders, we postulated that aberrant α‐syn expression in FCD IIb and TSC lesions might arise from the disrupted mTOR‐GLT‐1 regulatory axis.

Briefly, with cortical lesions collected from patients with FCD IIb and TSC, along with the adoption of FCD models generated by in utero X‐ray irradiation, we investigated the contribution of the mTOR‐GLT‐1 axis in α‐syn pathology and the epileptogenic progress in FCD IIb and TSC.

## 2 Materials and Methods

### 2.1 Human Specimens

Patients with drug‐resistant epilepsy attributed to FCD IIb and TSC were diagnosed in accordance with the consensus guidelines established by the International League Against Epilepsy [24]. Cortical lesions of FCD IIb and TSC were identified with MRI, stereo‐EEG recording, and subsequently confirmed by postoperative pathological examination after dissection. Tissues were fixed in 4% paraformaldehyde (PFA) immediately following dissection and embedded in paraffin blocks afterwards for immunostaining. This study enrolled 7 patients with FCD IIb and 5 patients with TSC. Due to ethical restrictions prohibiting the collection of healthy cortical tissue, inclusion criteria for the control group were established based on previous studies [25, 26]. Control cortical tissues were obtained from peri‐tumoral regions of brain tumor patients with no history of neurological disorders or epilepsy. Postoperative pathological analysis confirmed the absence of reactive astrocyte hyperplasia, necrosis, or inflammation. A total of 8 control subjects met these criteria and were included as controls. Informed consent was obtained from all patients or their legal guardians prior to tissue collection, and written consent forms were duly signed. The clinical characteristics of all the patients enrolled in the study are detailed in Tables 1, 2, 3.

**TABLE 1:** The detailed information of patients with FCD IIb.

No.	Gender	Diseases	Age at surgery (years)	Age of seizure (years)	Epilepsy duration (years)	Location	Seizure type	Seizure frequency (/month)	Anti‐seizure mediations	Engel's class	Application
1	F	FCD IIb	8.4	6.4	2.0	F	GTS	120	LEV, OXC	I	IHC
2	M	FCD IIb	4.0	1.0	3.0	LF	FBTCS	105	LEV, LTG, VPA, TPM	I	IHC, IF
3	F	FCD IIb	13.3	10.3	3.0	RF	GTS	75	LEV, OXC	I	IHC, IF
4	F	FCD IIb	15.0	12.0	3.0	LF	GTCS	120	OXC, NZP, LEV	I	IHC, IF
5	M	FCD IIb	19.0	3.0	16.0	LT	FIAS	1	VPA, OXC	III	IHC, IF
6	M	FCD IIb	6.0	5.1	0.9	T	FBTCS	600	OXC, LEV, NZP	I	IHC
7	M	FCD IIb	21.0	9.0	12.0	RT	GAS	2	VPA, CBZ, LTG	I	IHC

*Note:* F = female; M = male.

Abbreviations: CBZ: carbamazepine; F: frontal; FBTCS: focal to bilateral tonic–clonic seizure; FIAS: focal impaired awareness seizures; GAS: generalized absence seizure; GTCS: generalized tonic–clonic seizure; GTS: generalized tonic seizure; IF: immunofluorescence; IHC: immunohistochemistry; LEV: levetiracetam; LF: left frontal; LT: left temporal; LTG: lamotrigine; NZP: nitrazepam; OXC: oxcarbazepine; RF: right frontal; RT: right temporal; T: temporal; TPM: topiramate; VPA: valproate; WB: western botting.

**TABLE 2:** The detailed information of patients with TSC.

No.	Sex	Diseases	Age at surgery (years)	Age of seizure (years)	Epilepsy duration (years)	Location	Seizure type	Seizure frequency (/month)	Antiseizure mediations	Engel's class	Application
1	F	TSC	8.0	3.0	5.0	F	GTCS	45	VPA, LEV, CBZ, PB, NZP	IV	ICH, IF
2	F	TSC	5.0	2.0	3.0	LF	FIAS	1.0	VGB, LTG	I	IHC, IF
3	M	TSC	14.0	9.0	5.0	RF	GTCS	4.0	VPA	I	IHC, IF
4	M	TSC	11.8	11.0	0.8	LT	GTCS	5.0	OXC	I	IHC, IF
5	M	TSC	15.0	14.8	0.2	T	GTCS	1.0	OXC	II	IHC

*Note:* F = female; M = male.

Abbreviations: CBZ: carbamazepine; F: frontal; FBTCS: focal to bilateral tonic–clonic seizure; FIAS: focal impaired awareness seizures; GTCS: generalized tonic–clonic seizure; IF: immunofluorescence; IHC: immunohistochemistry; LEV: levetiracetam; LF: left frontal; LT: left temporal; LTG: lamotrigine; NZP: nitrazepam; OXC: oxcarbazepine; PB: phenobarbital; RF: right frontal; T: temporal; VGB: vigabatrin; VPA: valproate; WB: western botting.

**TABLE 3:** The detailed information of control cortex from patients with brain tumor.

No.	Sex	Surgery at age (years)	Disease	Application
1	M	6	RF primitive neuroectoblastoma	IHC
2	M	9	RF primitive neuroectoblastoma	IHC
3	M	16	LF glioma	IHC
4	M	5	RT glioma	IHC
5	M	11	LT glioma	IHC
6	M	14	RT glioma	IHC
7	F	15	LT glioma	IHC
8	F	19	RT glioma	IHC

*Note:* F = female; M = male.

Abbreviations: IHC: immunohistochemistry; LF: left frontal; LT: left temporal; RF: right frontal; RT: right temporal.

### 2.2 Animals

Sprague–Dawley (SD) rats and adult C57BL/6J mice were obtained from the Animal Facility of Army Medical University and housed at Xinqiao Hospital under standard conditions: a temperature of approximately 23°C, relative humidity of approximately 60%, a 12‐h light/dark cycle, and ad libitum access to food and water [27]. To generate the FCD model offspring, pregnant SD rats at 17.5 days of gestation were randomly subjected to X‐ray irradiation at a dose of 145 Gy for 60 s [28]. Pregnant rats in the control group were similarly handled but received 0 Gy irradiation for 60 s to produce normal offspring. All newborn pups were weaned 21 days after birth. Adult C57BL/6J mice with either gender aged 2–3 months were randomly assigned for preformed fibrils of α‐syn (α‐syn PFF) (ACRO, ALN‐M51H3) or PBS injection in the study.

### 2.3 RT‐qPCR

Total RNA was extracted from each sample using TRIzol reagent (Invitrogen, Cat. #15596026CN, CA) according to the manufacturer's protocol. RNA concentration and purity were assessed by measuring the absorbance at 260/280 nm using a NanoDrop spectrophotometer. Samples exhibiting an A260/A280 ratio between 1.8 and 2.1 were selected for subsequent experiments. PrimeScript RT Reagent Kit with gDNA Eraser (Takara Bio, Cat. #RR047A, Japan) was used to synthesize cDNA. qPCR was performed with a TB Green Premix Ex Taq II kit (Takara Bio, Cat. #RR820A, Japan) on a Bio‐Rad CFX96 instrument. The RT reagent kit (RR047A, Takara) includes gDNA Eraser to remove genomic DNA, which avoids gDNA contamination during cDNA synthesis and RT‐qPCR.

All primer sequences are provided in Supporting Information Table S1.

The PCR cycle conditions were as follows: initial annealing at 95°C for 30 s (1 cycle), followed by 49 cycles of 95°C for 5 s and 60°C for 40 s, with an extension at 95°C for 5 s. Each quantitative PCR was performed in triplicate, with at least two independent experiments conducted to determine the expression of the *mTOR*, *α‐syn* or *Glt‐1* mRNA relative to *Gapdh* according to the ΔCt methods [29], indicating that a lower ΔCt value would represent higher gene expression levels, whereas a higher ΔCt value demonstrates a lower gene expression levels.

### 2.4 Western Blotting

Protein was extracted with RIRA lysate buffer (Beyotime, Cat. P0013B, China) containing phosphatase and protease inhibitors, and the protein concentration was determined with a BCA kit (Beyotime, Cat. P0009, China). Proteins (40 μg per lane) were separated on 6% (Beyotime, Cat. P0448S, China) or 12% SDS‐PAGE gels (Beyotime, Cat. P0811S, China) and transferred to PVDF membranes with 0.45 μm pores. The membrane was blocked with 10% skim milk for 1 h, followed by an overnight incubation with primary antibody at 4°C. The membranes were then washed three times with TBST and incubated with a secondary antibody at 37°C for an hour. The signal was detected using a chemiluminescence solution (Thermo Scientific, Cat. 32106, USA) and the optical density (OD) values for each protein band were quantified with ImageJ software. Antibodies used for western blotting and their concentrations are listed as follows in the Table S2.

### 2.5 Immunostaining

Following fixation in 4% PFA, tissues were processed through a graded ethanol series (50%–100%), cleared in xylene, and embedded in paraffin blocks. Sections of 4 μm thickness were then prepared for histological analysis. Once the processes of dewaxing and rehydration were complete, the sections were subjected to heat‐induced antigen retrieval by microwaving in boiling citrate buffer for 30 min, and then allowed to cool to ambient temperature. The sections were incubated with 0.3% hydrogen peroxide at 37°C for 1 h to block endogenous peroxidase activity and incubated with corresponding primary antibodies (Table S2). Sections were incubated with corresponding HRP‐conjugated secondary antibodies for 1 h at 37°C after being washed three times in PBS. IHC staining was developed using a 3,3′‐diaminobenzidine (DAB) substrate kit (cat.: ZLI‐9017, Zhongshan Golden Bridge), followed by counterstaining with hematoxylin for 10 min. Differentiation was performed in 0.5% acid alcohol (1% HCl in 70% ethanol) for 30 s prior to bluing. Images were obtained with an inverted bright‐field microscope (BX63, Olympus, Japan). The primary antibodies used for immunohistochemistry (IHC) are listed in Table S2.

For immunofluorescent staining, paraffin‐embedded sections were blocked in 10% goat serum after deparaffinization and antigen retrieval and incubated with corresponding primary antibodies overnight at 4°C. After washing three times with PBS, the sections were incubated with corresponding secondary antibodies for 1 h at 37°C. Sections were subsequently nuclear stained by Hoechst 33258 and photographed by a confocal microscope (LSM880, ZEISS, Germany). The specific antibodies used for IHC are listed in Table S2.

For frozen sections staining, brain tissues were collected and fixed with 4% PFA overnight, then these tissues were dehydrated in 30% sucrose. Brains were embedded in optimal cutting temperature compound (O.C.T. Compound, SAKURA, 4583, USA) and sliced coronally 20 μm on a cryostat microtome (MS 1850, Leica, Wetzlar, Germany). Floating sections were blocked with 7% bovine serum albumin (BSA) and 0.2% Triton‐X 100 for 2 h at room temperature and then incubated with primary antibodies overnight at 4°C and then sequentially incubated with corresponding secondary antibodies for 1 h at room temperature. Nuclear stained by Hoechst 33258 and photographed by a confocal microscope (LSM880, ZEISS, Germany).

### 2.6 Evaluation of Immunohistochemical (IHC) Immunoreactivity

The immunoreactivity of mTOR, p‐mTOR, α‐syn, p‐α‐syn, and GLT‐1 was assessed using ImageJ according to a previous method [30]. Briefly, brightness and contrast were uniformly adjusted across all panels, and no repeated fields of view were found at the center of the lesions (400× magnification, 0.3481 mm × 0.2621 mm width). Three slices per sample were chosen, with three fields from each slice selected. The values of OD from these nine areas were averaged. Two researchers evaluated immunoreactivity in a double‐blinded manner.

For the cell number and synaptic puncta density quantification, at least three representative fields (40× and oil 63×) were randomly acquired from layer V of the cortex using ImageJ according to previous literature [31].

### 2.7 Cannula Implantation

Rats were anesthetized with isoflurane (induction: 5%, maintenance: 1.5%–2% in O_2_) to achieve surgical anesthesia (loss of pedal reflex) for stereotaxic implantation. A guide cannula (RWD) was stereotactically positioned in the left lateral ventricle for α‐syn injection at coordinates relative to bregma: 1.8 mm posterior, 0.72 mm lateral, and 3.5 mm ventral from the skull surface. Passive cerebrospinal fluid efflux through the cannula upon implantation provides primary confirmation of correct intraventricular placement, attributable to intrinsic intracranial pressure gradient. For definitive verification, a microsyringe was connected to apply negative pressure. Aspiration of clear cerebrospinal fluid confirmed cannula positioning within the lateral ventricle [32]. Failure to obtain cerebrospinal fluid or aspiration of hemorrhagic fluid indicated placement in parenchymal or vascular compartments. Animals meeting either exclusion criterion were immediately withdrawn from the study. α‐syn (300 μM, 250 μL) was infused into the lateral ventricle through the cannula at 0.5 μL/min (total volume ≤ 10% of estimated CSF capacity) [33].

### 2.8 EEG Recording

For EEG recording, electrodes were stereotactically implanted in the bilateral frontal cortex (coordinates relative to bregma: +4.0 mm AP, ±4.0 mm ML, 0.0 mm DV) of FCD rats under the anesthetization with isoflurane in consistent with previous methods [34], with cerebellar vermis and bilateral olfactory bulbs serving as reference sites. All implants were secured to the skull using cyanoacrylate adhesive (Loctite 401). Following surgery, animals received buprenorphine analgesia and were allowed 72‐h recovery before EEG recording.

Pentylenetetrazole (PTZ) (40 mg/kg) was intraperitoneally injected to induce seizures in FCD animals according to previous methods [35, 36, 37]. EEG was recorded with RM6240E (Chengdu instrument factory) at a sampling rate of 1 kHz and continuously recorded for 2 h after PTZ injection. Based on established electrographic criteria [38, 39], seizures were defined as ictal events lasting ≥ 5 s with amplitude exceeding twice the baseline of EEG activity. EEG data was processed using MATLAB R2023a.

### 2.9 Injection of α‐Syn PFF

Adult C57BL/6J mice were anesthetized with isoflurane (induction: 5%, maintenance: 1.5%–2% in O_2_) to achieve surgical anesthesia (loss of pedal reflex) for stereotaxic injection. Thereafter, α‐syn PFF (2 μL, 5 mg/mL) (ACRO, Cat.ALN‐M51H3, China) was injected at a rate of 0.5 μL/min in the prefrontal cortex (coordinates: 2.4 mm, +0.4 mm, 1.4 mm) of C57 mice, and the needle was kept in the injected place for 5 min after injection to ensure the diffusion of α‐syn PFF in the cortex, and an equal volume of PBS was used as control according to the previous studies [40, 41].

### 2.10 Statistical Analysis

Data acquisition and analysis were done blindly and presented as means ± SEM. Statistical methods, the number of replicates and number of animals or specimens were indicated as needed. For comparisons between two independent groups, an unpaired two‐tailed *t*‐test was used. One‐way ANOVA followed by Dunnett's multiple comparisons test was used for comparisons in more than two groups. Spearman's rank correlation test was used for correlation analysis, where samples for the paired indicators originated from the same specimens. Chi‐square test was used for comparison of the mortality and survival rates between two groups. GraphPad prism 10 software was used for data plotting and analysis. The same set of biological samples was used consistently for all comparisons and normalized to the CTX group. *p* < 0.05 was considered statistically significant.

## 3 Result

### 3.1 The Expression of mTOR and p‐mTOR in the Cortical Lesions of Patient With FCD IIb and TSC

**FIGURE 1:**
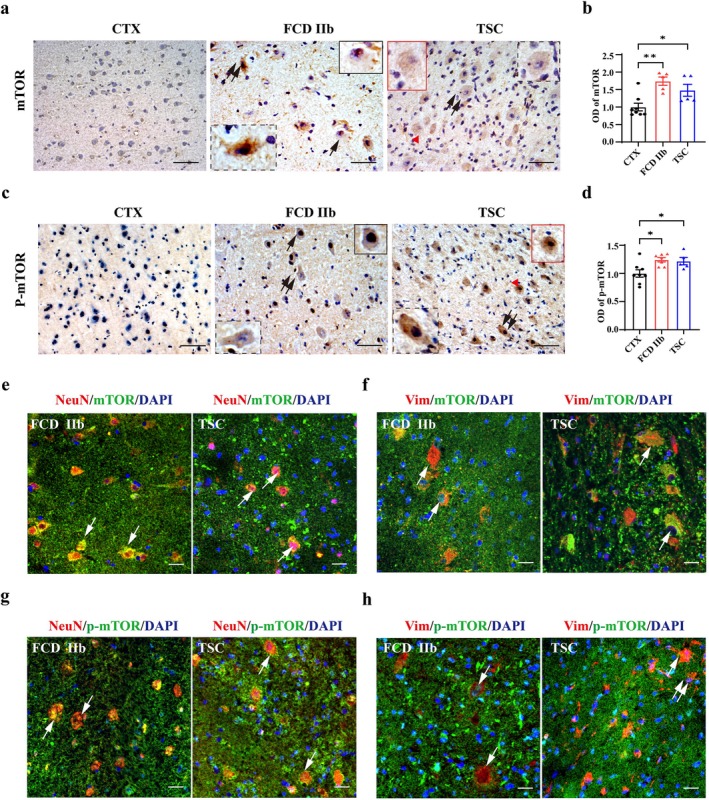
The expression patterns of mTOR and p‐mTOR in cortical lesions from patients with FCD IIb and TSC. Representative images showing mTOR was rarely observed in the neurons of CTX, whereas was enhanced in the cytoplasm of DNs (double arrows and insets with dotted border), BCs (arrow and insets with solid border) and GCs (red arrowhead and inset) in cortical lesions of patients with FCD IIb and TSC. (b) Statistics showing the OD value of mTOR was significantly increased in the cortical lesions of patients with FCD IIb and TSC, respectively (*p < 0.05, **p < 0.01, one‐way ANOVA test, *n* = 5–8 for each group). (c) Representative images showing weak p‐mTOR signaling in the neurons of CTX, whereas was significantly enhanced in the cytoplasm of DNs (double arrows and insets with dotted border), BCs (arrow and insets with solid border) and GCs (red arrowhead and inset) in cortical lesions of patients with FCD IIb and TSC. (d) Statistical analysis showing that the average OD value of p‐mTOR was significantly increased in the cortical lesions of patients with FCD IIb and TSC (*p < 0.05, one‐way ANOVA test, *n* = 5–8 for each group). (e, f) Representative images showing mTOR was expressed in NeuN‐positive neurons (e, arrows), vimentin‐positive BCs and GCs (f, arrows) in the cortical lesions of patients with FCDIIb and TSC, respectively. (g, h) Representative images showing p‐mTOR was expressed in NeuN‐positive neurons (g, arrows), vimentin‐positive BCs and GCs (h, arrows) in the cortical lesions of patients with FCDIIb and TSC, respectively. Scale bars: 50 μm for figure a,c, 20 μm for figure e–h.

IHC experiments were performed to evaluate the expression level of mTOR and p‐mTOR in the cortical lesions of patients with FCD IIb and TSC. The immunoreactivity of mTOR and p‐mTOR was relatively weak in the neurons of control cortex (CTX). In contrast, both the immunoreactivity of mTOR and p‐mTOR were substantially strengthened in the dysmorphic neurons in FCD IIb and TSC lesions, including DNs (double arrows), BCs (arrow), and GCs (red arrowhead), as shown in the representative images and insets (Figure 1a,c). Evaluation of the intensity of mTOR immunoreactivity revealed that both the OD value of mTOR (*p < 0.05, **p < 0.01, *n* = 5–8 for each group) and p‐mTOR (*p < 0.05, *n* = 5–8 for each group) were increased in the cortical lesions of patients with FCD IIb and TSC, respectively (Figure 1b,d). Double immunofluorescent staining results revealed that mTOR was substantially expressed in NeuN‐positive DNs, and vimentin‐positive BCs and GCs in the cortical lesions of patients with FCD IIb and TSC (arrows, Figure 1e,f). Similarly, p‐mTOR was substantially expressed in NeuN‐positive DNs (arrows, Figure 1g) and vimentin‐positive BCs and GCs (arrows, Figure 1h). Additionally, vimentin‐positive cells with astrocyte morphology were observed in TSC lesions (double arrows, Figure 1h). These results suggested that the mTOR signaling pathway was significantly activated in the cortical lesions of patients with FCD IIb and TSC.

### 3.2 Rapamycin Restored mTOR/p‐mTOR Ratio in Cortical Lesions of FCD Animals

**FIGURE 2:**
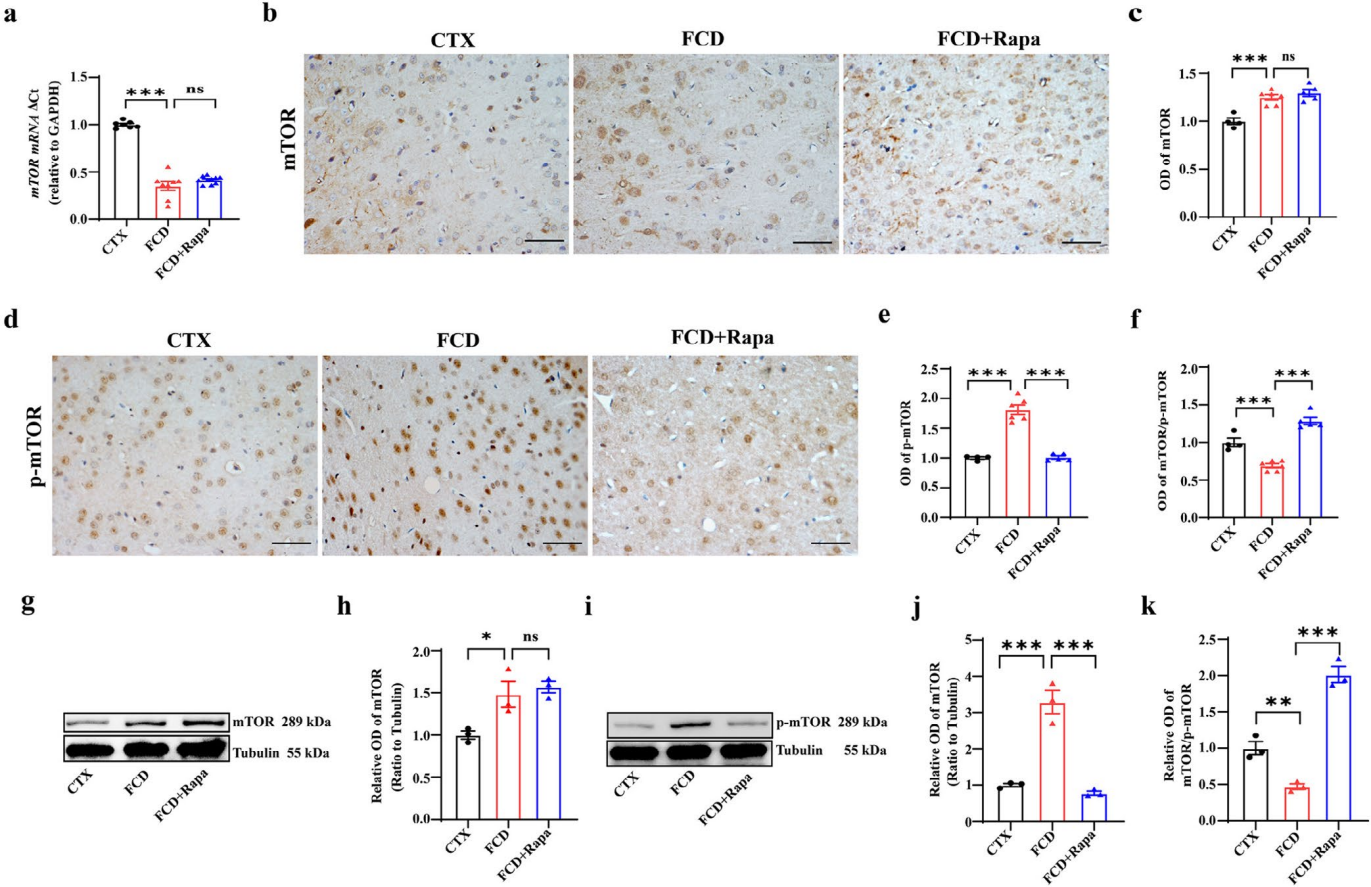
Rapamycin restored mTOR/p‐mTOR ratio in cortical lesions of FCD animals. (a) RT‐PCR results showing that the mTOR mRNA was significantly increased in the cortical lesions of FCD rats, whereas it was not altered by rapamycin (Rapa) treatment (ns, nonsignificant, *p < 0.05, one‐way ANOVA test, *n* = 7–10 for each group). (b) Representative IHC images showing weak and moderate mTOR immunoreactivity in cortical layer V neurons across control, FCD, and FCD + Rapa treated animals. (c) Statistics showing the OD value of mTOR was increased in the cortical lesions of FCD rats, but not affected by Rapa (ns, nonsignificant, ***p < 0.001, one‐way ANOVA test, *n* = 4–6 for each group). (d) Representative IHC images showing moderate p‐mTOR immunoreactivity in cortical layer V neurons of control animals and significantly intensified FCD animals, whereas was weakened by Rapa treatment. (e) Statistical analysis showing the OD value of p‐mTOR was significantly increased in FCD animals, but rescued by Rapa treatment (***p < 0.001, one‐way ANOVA test, *n* = 4–6 for each group). (f) Statistics showing the mTOR/p‐mTOR ratio was significantly compromised in FCD animals; however, was restored by Rapa treatments (***p < 0.001, one‐way ANOVA test, *n* = 4–6 for each group). (g, h) Representative gel blots and corresponding statistics showing that the expression of mTOR protein (approximately 289 kDa) was significantly increased in the homogenates from the cortical lesions of FCD rats, whereas was not altered by Rapa treatment, and tubulin (approximately 55 kDa) was used as internal control (ns, nonsignificant, *p < 0.05, one‐way ANOVA test, *n* = 3 for each group). (i, j) Representative gel blots and corresponding statistics showing that the expression of p‐mTOR protein (approximately 289 kDa) was significantly increased in the homogenates from the cortical lesions of FCD rats, whereas were efficiently rescued by Rapa treatment. Tubulin was used as internal control (***p < 0.001, one‐way ANOVA test, *n* = 3 for each group). (k) Statistical analysis showing the mTOR/p‐mTOR ratio was significantly compromised in FCD animals; however, was restored by Rapa treatment (**p < 0.01, ***p < 0.001, one‐way ANOVA test, *n* = 3 for each group). Scale bars: 50 μm for figure b,d.

Rapamycin is a highly selective inhibitor of mTOR [42]. With the utilization of FCD models generated by in utero X‐ray irradiation, we investigated the effect of continuous daily injection of rapamycin (Rapa) (6 mg/kg, i.p.) for 7 days on the expression of mTOR, p‐mTOR in FCD rats, respectively [43]. Consistent with the alterations of mTOR in cortical lesions of patients with FCD IIb and TSC, RT‐PCR results showed that the expression of *mTOR* RNA was increased in the cortical lesions of FCD animals, whereas Rapa did not efficiently restored the expression of *mTOR* RNA (Figure 2a, ns, nonsignificant, *p < 0.05, *n* = 7–10 for each group). Representative images of IHC staining and relative statistics further confirmed that the immunoreactivity of mTOR (ns, nonsignificant, ***p < 0.001, *n* = 4–6 for each group), p‐mTOR (***p < 0.001, *n* = 4–6 for each group) were consistently increased in the cortical layer V neurons in FCD animals, and efficiently restored by Rapa treatment (Figure 2b–e). Notably, the mTOR/p‐mTOR ratio was significantly reduced in the cortical lesions of FCD animals due to the intensified immunoreactivity in the cellular cytoplasm and nuclei, however, was effectively rescued by Rapa treatment (Figure 2f, ***p < 0.001, *n* = 4–6 for each group). Additionally, gel electrophoresis from the homogenates of cortical lesions of FCD animals confirmed that Rapa was not altered the mTOR protein (ns, nonsignificant, *p < 0.05, *n* = 3 for each group), but it efficiently rescued the increased p‐mTOR protein (approximately 289 kDa) (***p < 0.001, *n* = 3 for each group) in FCD animals with tubulin used as internal control, as shown in the representative gel blots and corresponding statistics (Figure 2g–j). Furthermore, the mTOR/p‐mTOR ratio was significantly reduced in the cortical lesions of FCD animals, indicating a relatively greater increase in p‐mTOR expression. However, Rapa efficiently rescued the reduced mTOR/p‐mTOR ratio in FCD animals (Figure 2k, **p < 0.01, ***p < 0.001, *n* = 3 for each group), suggesting that Rapa could efficiently inhibit over‐activated mTOR signaling in FCD animals.

### 3.3 Rapamycin Rescued p‐α‐Syn Accumulation Induced Excitotoxity in Cortical Lesions of FCD Animals

**FIGURE 3:**
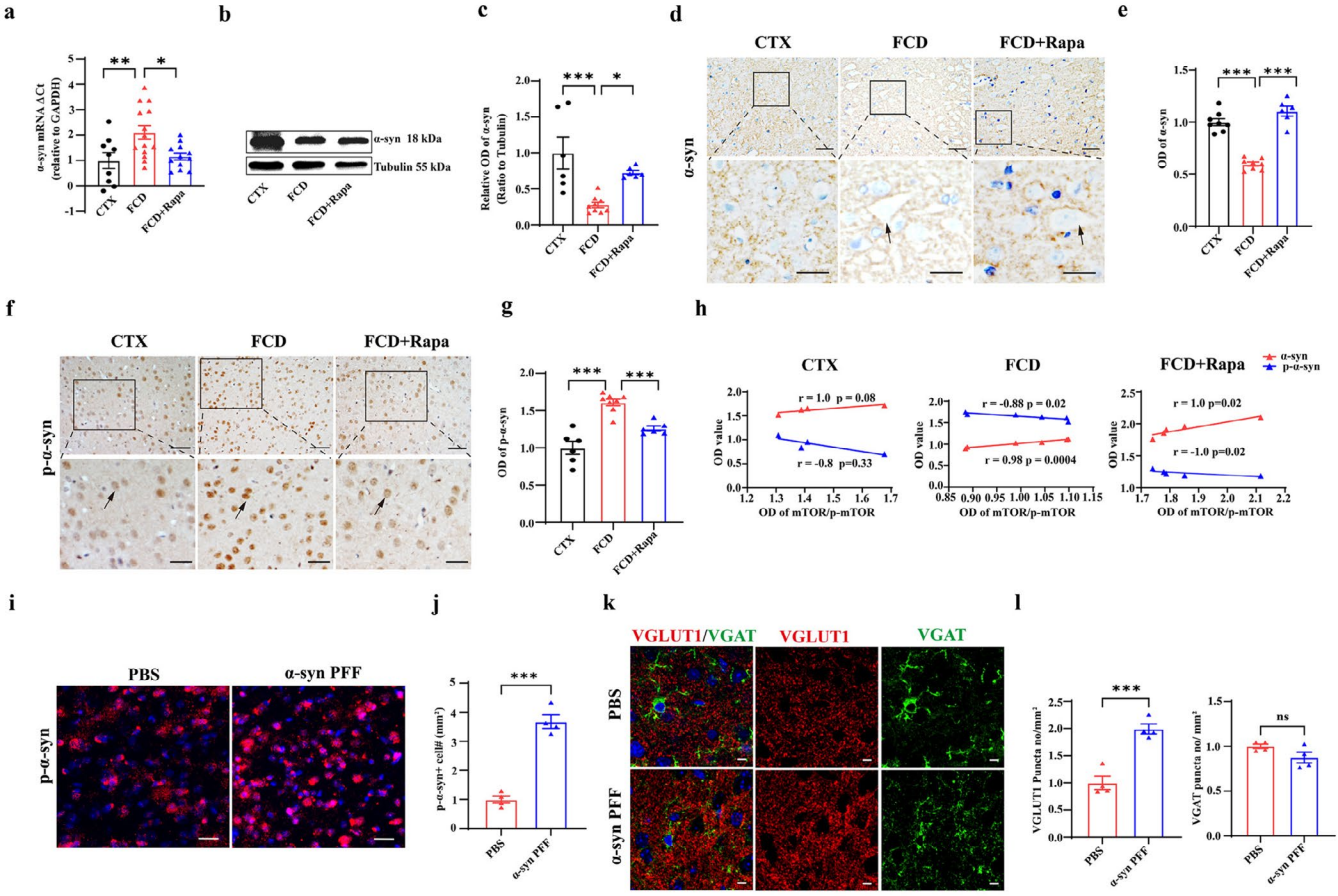
Rapamycin rescued p‐α‐syn accumulation induced excitotoxicity in cortical lesions of FCD animals. (a) RT‐PCR results showing that the expression level of α‐syn mRNA was significantly reduced in cortical lesions of FCD rats, whereas it was up‐regulated by Rapa treatment (*p < 0.05, **p < 0.01, one‐way ANOVA test, *n* = 9–14 for each group). (b, c) Representative gel blots and corresponding statistics showing that the expression of α‐syn protein (approximately 18 kDa) was significantly reduced in the homogenates from cortical lesions of FCD rats, whereas it was efficiently rescued by Rapa treatment. Tubulin (approximately 55 kDa) was used as internal control (*p < 0.05, ***p < 0.001, one‐way ANOVA test, *n* = 6–9 for each group). (d, e) Representative images and corresponding statistics showing intense α‐syn expression in the neuropils of cortical layer V neurons in control animals. The immunoreactivity of α‐syn was significantly reduced in the cortical lesions of FCD animals, whereas it was intensified after Rapa treatment (***p < 0.001, one‐way ANOVA test, *n* = 6–8 for each group). Arrows indicated normal, DN‐like cells in control, FCD and FCD + Rapa treated animals, respectively in the bottom higher‐magnified images. Scale bars: 50 μm for upper panel, 25 μm for bottom panel. (f, g) Representative images and corresponding statistics showing intense p‐α‐syn accumulation in the neuronal cytoplasm and nucleus of cortical lesions from FCD rats; however, Rapa treatment effectively down‐regulated the accumulation of p‐α‐syn in FCD rats (***p < 0.001, one‐way ANOVA test, *n* = 6–8 for each group). Arrows indicated the accumulation of p‐α‐syn in the representative cells. Scale bars: 50 μm for upper panel, 25 μm for bottom panel. (h) Correlation analysis showing that mTOR/p‐mTOR ratio was positively correlated with the expressional level of α‐syn, but negatively correlated with the expressional level of p‐α‐syn both in FCD and FCD + Rapa groups (Spearman's correlation test, *n* = 4–6 for each group). (i, j) Representative immunofluorescent images and statistics showing the increased accumulation of p‐α‐syn after injection of α‐syn preformed fibrils (α‐syn PFF) in the cortex (***p < 0.001, unpaired two‐tailed *t*‐test, *n* = 4 for each group). Scale bars: 20 μm. (k, l) Representative images and statistics showing that VGLUT1 puncta was significantly increased in α‐syn PFF treated animals, whereas the expression of VGAT was not altered (ns, nonsignificant, ***p < 0.001, unpaired two‐tailed *t*‐test, *n* = 4 for each group). Scale bars: 5 μm.

Subsequently, we investigated the effect of Rapa on the expression of α‐syn, p‐α‐syn in the cortical lesions of FCD animals. RT–PCR results showed that *α‐syn* mRNA was significantly reduced in the cortical lesions of FCD rats, whereas Rapa treatment efficiently increased the expression of *α‐syn* mRNA in FCD animals (Figure 3a, *p < 0.05, **p < 0.01, *n* = 9–14 for each group). Gel electrophoresis from the homogenates of the cortical lesions of FCD animals confirmed that Rapa effectively rescued the reduced α‐syn protein (approximately 18 kDa) in FCD animals with tubulin used as internal control (approximately 55 kDa), as shown in the representative gel blots and corresponding statistics (Figure 3b,c, *p < 0.05, ***p < 0.001, *n* = 6–9 for each group). Consistently, the immunoreactivity of α‐syn was reduced in the cortical layer V neuropils in FCD animals, whereas it was strengthened after Rapa treatment (Figure 3d,e, ***p < 0.001, *n* = 6–8 for each group), arrows indicated DNs with increased cell body in FCD lesions. For the effects of Rapa on the expression of p‐α‐syn, IHC staining and corresponding statistic showed that the intense accumulation of p‐α‐syn in cortical lesions was weakened in Rapa treated FCD animals (Figure 3f,g, ***p < 0.001, *n* = 6–8 for each group). Additionally, we analyzed the correlation of mTOR/p‐mTOR ratio with α‐syn, p‐α‐syn respectively. Statistics showed that mTOR/p‐mTOR ratio was positively correlated with the expression of α‐syn, while negatively correlated with the expression of p‐α‐syn in FCD animals either with or without Rapa treatment (Figure 3h), suggesting the modulatory role of mTOR signaling in the expression of α‐syn in FCD.

Previous studies have shown that α‐syn monomers tend to aggregate in structures of higher molecular weights leading to the formation of α‐syn oligomers, protofibrils, and eventually fibrils in neurodegenerative diseases [44]. Here in the study, we injected the protofibrils form of α‐syn, the α‐syn preformed fibrils (α‐syn PFF) into the frontal cortex according to methods modified from previous studies [40], to induce p‐α‐syn accumulation and investigate its effects on synaptic transmission afterwards. As the representative images and statistics showed, the number of p‐α‐syn positive cells was significantly increased in α‐syn PFF injected lesions (Figure 3i,j, ***p < 0.001, *n* = 4 for each group). Double immunostaining of the excitatory synaptic terminal marker, VGLUT1, and inhibitory synaptic marker, VGAT showed that the number of VGLUT1 positive puncta was significantly increased in α‐syn PFF injected lesions, whereas the number of VGAT positive inhibitory puncta was not affected (Figure 3k,l, ns, nonsignificant, ***p < 0.001, *n* = 4 for each group). In together, these results suggested that the deposition of p‐α‐syn may contribute to the imbalance of excitatory and inhibitory synaptic transmission in FCD lesions.

### 3.4 The Expressional Profiles of GLT‐1 in Cortical Lesions of Patients With FCD IIb and TSC

**FIGURE 4:**
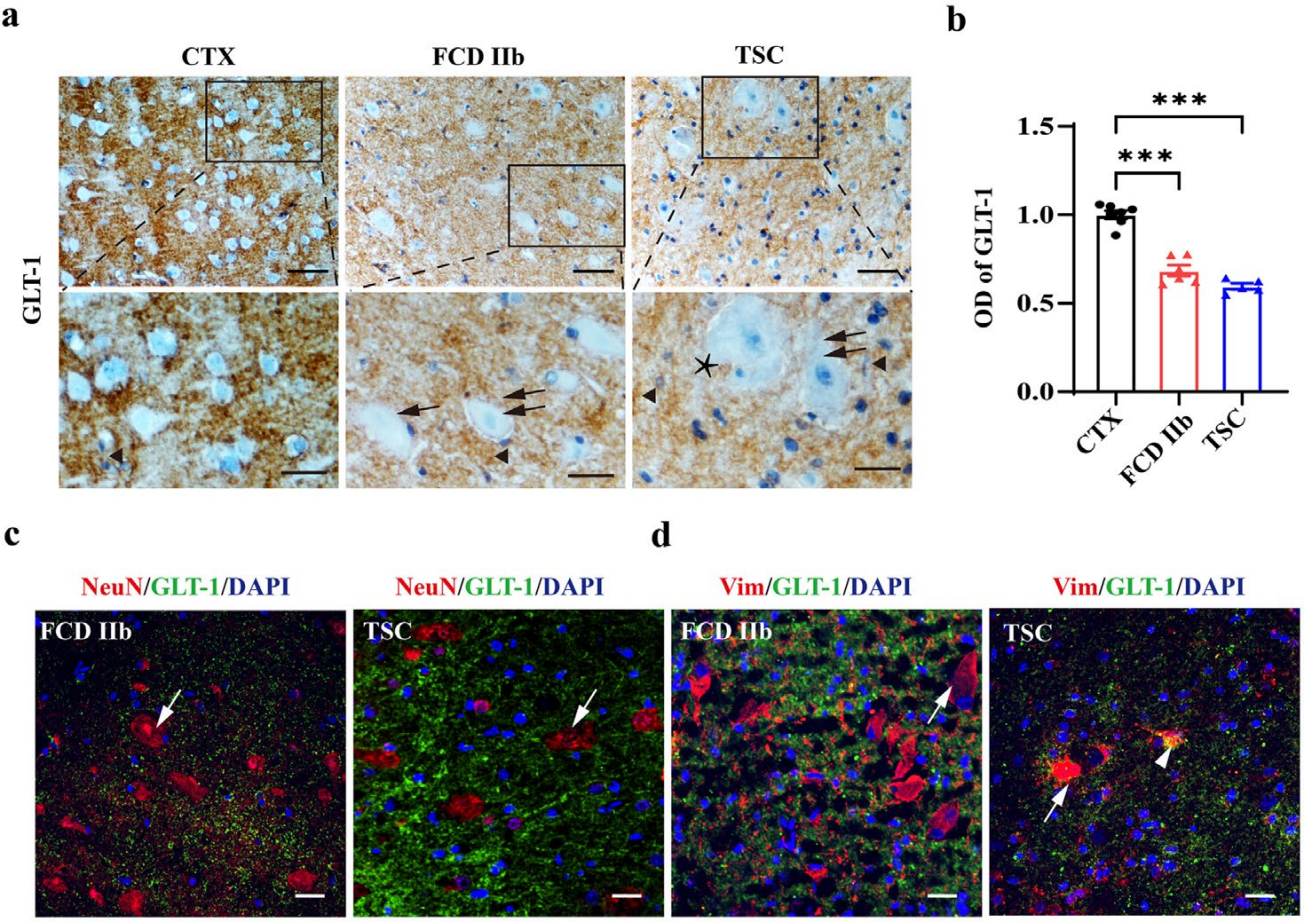
The expressional profiles of GLT‐1 in cortical lesions of patients with FCD IIb and TSC. Representative images showing strong and moderate immunoreactivity of GLT‐1 in control, FCD IIb, and TSC lesions. Double arrows indicated DNs with larger cell bodies in FCD IIb and TSC lesions, an arrow indicated BC in FCD IIb lesions, asterisk indicates a GC in TSC lesions, and arrowhead indicated GLT‐1 positive signals in cells with astrocyte morphology. Scale bars: 50 μm for upper panel, 25 μm for bottom panel. (b) Statistics showing that the OD value of GLT‐1 was reduced in the cortical lesions of patients with FCD IIb and TSC (***p < 0.001, one‐way ANOVA test, *n* = 5–7 for each group). (c, d) Representative immunofluorescent images showing GLT‐1 was primarily expressed in axonal terminals, but not in NeuN‐positive DNs (arrow, c) or vimentin‐positive BCs (arrows, d). Arrowhead indicated co‐expression of GLT‐1 with vimentin in the cytoplasm of an astrocyte‐like cell in TSC lesions. Scale bars: 20 μm.

IHC was adopted to evaluate the expression of GLT‐1 in the cortical lesions of patients with FCD IIb and TSC. As shown in the representative images, GLT‐1 was intensely expressed in the cortical neuropils from control subjects, while its immunoreactivity was significantly reduced in the neuropils of cortical lesions from patients with FCD IIb and TSC (Figure 4a,b, ***p < 0.001, *n* = 5–7 for each group). Notably, GLT‐1 was not detected in DNs (double arrows), BCs (arrow), and GCs (asterisk), whereas it was substantially expressed in astrocytes (arrowhead) in FCD IIb and TSC lesions (Figure 4a). Double immunofluorescent staining results further revealed that GLT‐1 was not expressed in NeuN‐positive DNs (arrow, Figure 4c) or vimentin‐positive BCs (arrow, Figure 4d) in cortical lesions from patients with FCD IIb and TSC, while it was expressed in the cytoplasm of a vimentin‐positive astrocyte in the representative image of TSC lesions (arrowhead, Figure 4d).

### 3.5 Rapamycin Positively Modulated the Expression of GLT‐1 in FCD Animals

**FIGURE 5:**
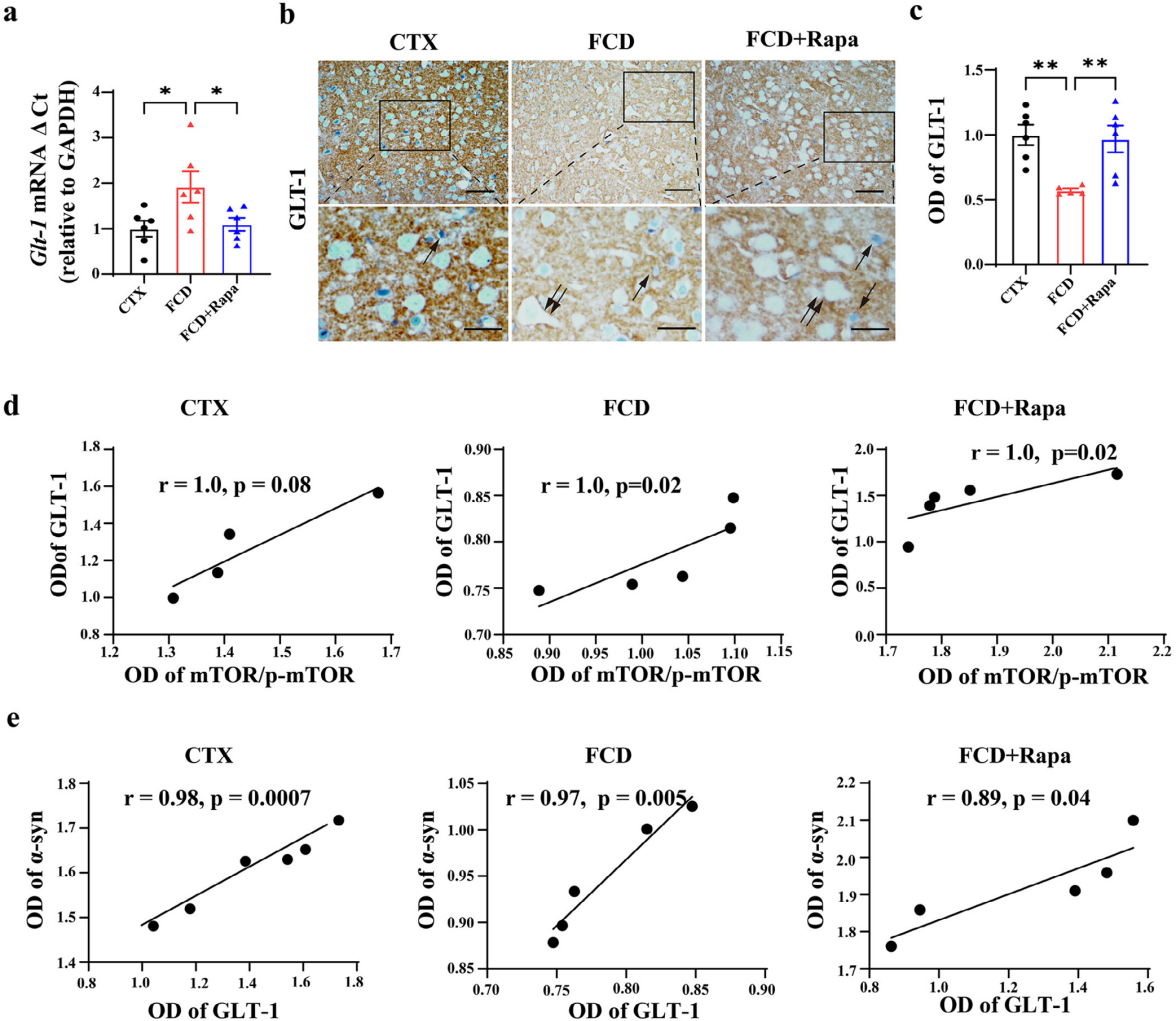
Rapamycin positively modulated the expression of GLT‐1 in FCD animals. (a) RT–PCR results showing that the expression of the *GLT‐1* mRNA was significantly reduced in the cortical lesions of FCD rats, whereas it was up‐regulated by Rapa treatment (*p < 0.05, one‐way ANOVA test, *n* = 6 for each group). (b) Representative immunohistochemistry images showing strong GLT‐1 immunoreactivity in the axonal terminals and astrocytes (arrow) of control cortex; relatively weak and moderate GLT‐1 immunoreactivity were detected in the cortical lesions of FCD and FCD + Rapa treated animals. Double arrows indicated DNs, and the arrow indicated astrocytes in the cortical lesions. Scale bar: 50 μm for upper panel, 25 μm for bottom panel. (c) Statistics showing the OD value of GLT‐1 was reduced in FCD animals and rescued by Rapa treatment (**p < 0.01, one‐way ANOVA test, *n* = 5–6 for each group). (d) Statistics showing that the mTOR/p‐mTOR ratio was positively correlated with the expression of GLT‐1 (Spearman's correlation test, *n* = 4–5 for each group). (e) Correlation analysis showing that the expression of GLT‐1 was positively correlated with the expression of α‐syn (Spearman's correlation analysis, *n* = 5–6 for each group).

Subsequently, we investigated the effects of Rapa on the expression of *Glt‐1* mRNA with RT‐PCR in FCD animals. RT‐PCR results showed that the expression of *Glt‐1* was significantly decreased in the cortical lesions of FCD animals. However, Rapa treatment efficiently increased the expression of *Glt‐1* (Figure 5a, *p < 0.05, *n* = 6 for each group). Next, we conducted IHC experiments to assess GLT‐1 protein expression in the cortical lesions of FCD rats after Rapa treatment. IHC staining revealed that GLT‐1 was intensely expressed in the neuronal axon terminals and astrocytes, but not in neuronal bodies of the control cortex. However, the immunoreactivity of GLT‐1 was relatively weak in the cortical lesions of FCD rats, whereas it was increased by Rapa treatment (Figure 5b,c, **p < 0.01, *n* = 5–6 for each group). Additionally, correlation analysis revealed that the mTOR/p‐mTOR ratio was positively correlated with the expression of GLT‐1 (Figure 5d), further suggesting the potential role of mTOR signaling pathway in regulating GLT‐1 mediated function. We also analyzed the correlation of GLT‐1 with α‐syn in FCD animals. Statistics revealed that the expression of GLT‐1 is positively correlated with the expression of α‐syn (Figure 5e), which emphasized the significance of mTOR/GLT‐1 axis in α‐syn related pathological mechanisms in FCD.

### 3.6 Ceftriaxone Treatment Ameliorated p‐α‐Syn Accumulation in Cortical Lesions of FCD Animals

β‐Lactam antibiotics, ceftriaxone (Cef) represent the most potent pharmacological GLT‐1 enhancer, demonstrating therapeutic efficacy across neurological disorders by up‐regulating GLT‐1 [45, 46, 47, 48, 49, 50, 51]. Therefore, we used Cef (200 mg/kg, i.p.) to selectively up‐regulate the expression of GLT‐1 in FCD animals, as shown by previous studies [52, 53]. RT–PCR showed that the expression of *Glt‐1* mRNA was significantly up‐regulated in the cortical lesions of the FCD rats after continuous injection of Cef for 7 days (Figure S1a, *p < 0.05, ***p < 0.001, *n* = 6 for each group), and the immunoreactivity of GLT‐1 (Figure S1b,c, ***p < 0.001, *n* = 7 for each group).

**FIGURE 6:**
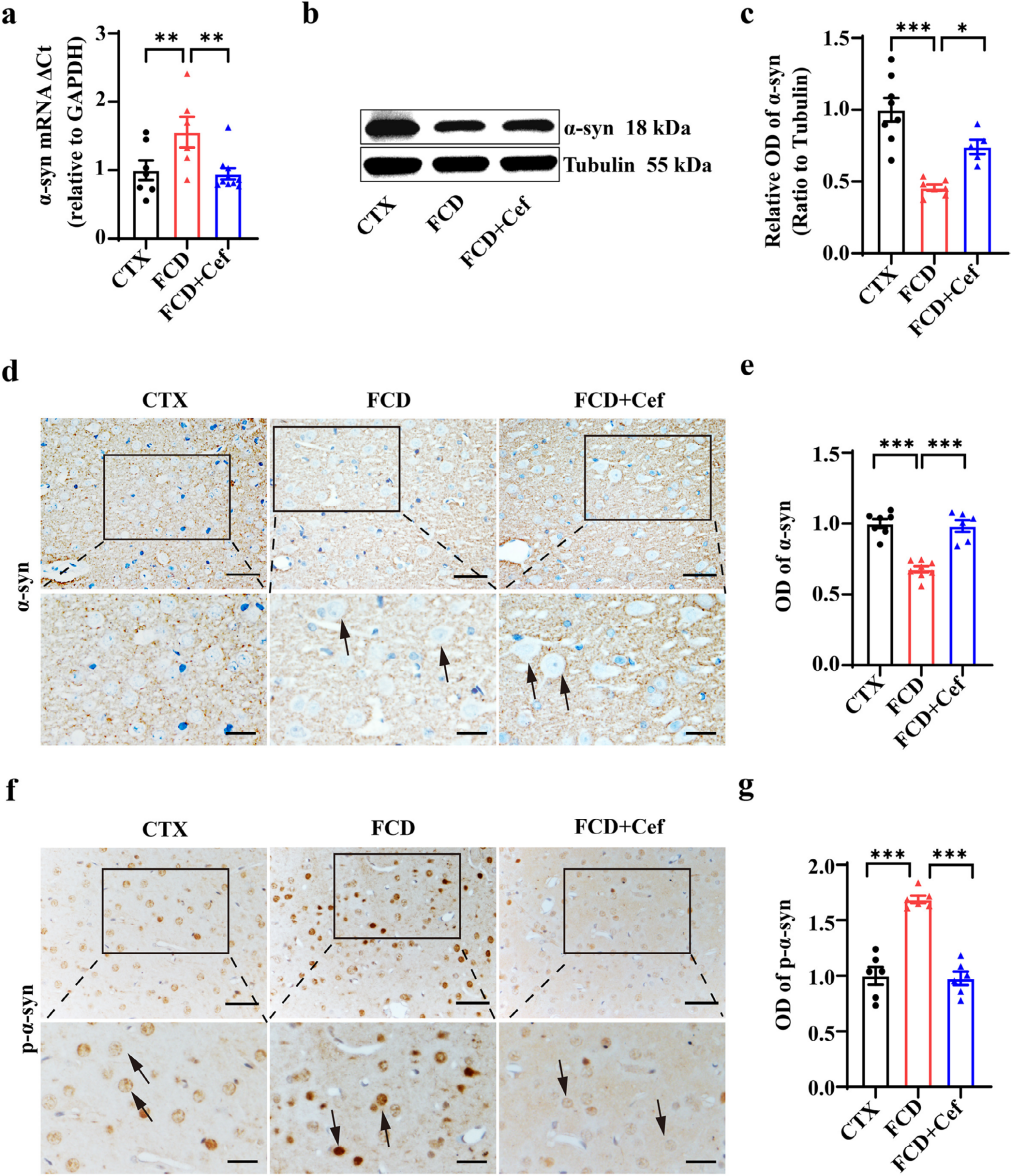
Ceftriaxone treatment ameliorated p‐α‐syn accumulation in cortical lesions of FCD rats. (a) RT–PCR results showing Cef treatment rescued the reduced expression of α‐syn mRNA in the cortical lesions of FCD animals (**p < 0.01, one‐way ANOVA test, *n* = 6–10 for each group). (b, c) Representative gel blots and relative statistics showing that the expression of α‐syn protein (18 kDa) was reduced in cortical lesions of FCD animals, whereas it was rescued by Cef treatment (*p < 0.05, ***p < 0.001, one‐way ANOVA test, *n* = 5–8 for each group). Tubulin was used as internal control (55 kDa). (d, e) Representative IHC images and statistics showing that the immunoreactivity of α‐syn in neuropils was reduced in the cortical lesions of FCD rats, whereas it was up‐regulated after Cef treatment (***p < 0.001, one‐way ANOVA test, *n* = 6–8 for each group). Arrow indicated that α‐syn immunoreactivity was absent in the DNs of FCD lesions. (f, g) Representative IHC images and statistics showing that the enhanced immunoreactivity of p‐α‐syn in the cortical lesions of FCD animals was reduced by Cef treatment (***p < 0.001, one‐way ANOVA test, *n* = 6 for each group). Arrow indicated p‐α‐syn accumulation in the cytoplasm and nuclei. Scale bars: 50 μm for upper panel, 25 μm for bottom panel in figure d,f.

We subsequently investigated the effects of Cef treatment on the level of α‐syn and p‐α‐syn in the cortical lesions of FCD rats. RT–PCR results revealed that the expression of *α‐syn* mRNA in the cortical lesions of FCD rats was significantly increased after Cef treatment (Figure 6a, **p < 0.01, *n* = 6–10 for each group). Western blotting confirmed that the expression of α‐syn protein was also significantly up‐regulated by Cef in FCD animals, as shown by the representative gel blots and relative statistics (Figure 6b,c, *p < 0.05, ***p < 0.001, *n* = 5–8 for each group). IHC staining results indicated that the immunoreactivity of α‐syn in neuropils was weakened in the cortical lesions of the FCD rats, whereas it was strengthened after treatment with Cef (Figure 6d,e, ***p < 0.001, *n* = 6–8 for each group), with arrows indicating DNs in the FCD lesions. Consistently, the intensified immunoreactivity of p‐α‐syn in FCD rats was reduced by Cef treatment (Figure 6f,g, ***p < 0.001, *n* = 6 for each group). These results suggested that the α‐syn and p‐α‐syn could be effectively regulated by GLT‐1 in FCD animals.

### 3.7 Ceftriaxone Treatment Ameliorated Seizure Activities Induced by PTZ in FCD Animals

**FIGURE 7:**
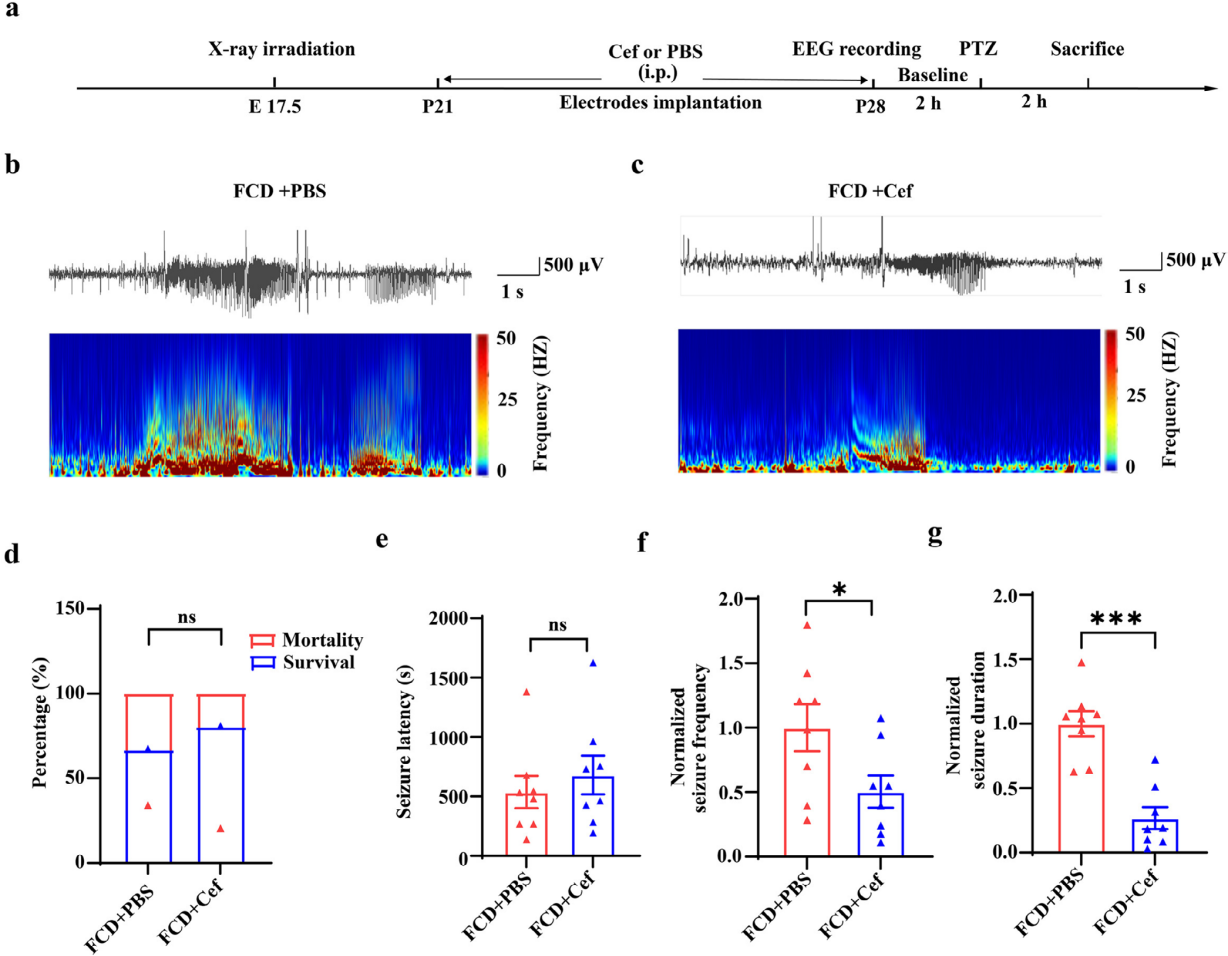
Ceftriaxone treatment ameliorated seizure activities induced by PTZ in FCD animals. (a) Experimental paradigm. (b, c) Representative EEG traces and corresponding time‐frequency diagrams recorded from the frontal cortex of PBS‐ and Cef‐treated FCD animals during seizure discharges after PTZ injection. (d) Statistics showing that PTZ induced mortality was not affected by Cef treatment in FCD animals (ns, nonsignificant, two‐tailed chi‐square test, *n* = 10–12 for each group). (e) Statistics showing that the latency of PTZ‐induced seizures was not affected by Cef (ns, nonsignificant, unpaired two‐tailed *t*‐test, *n* = 8 for each group). (f, g) Statistics showing that Cef treatment significantly reduced both seizure frequency and duration in FCD animals (*p < 0.05, ***p < 0.001, unpaired two‐tailed *t*‐test, *n* = 8 for each group).

Next, we investigated the effects of Cef treatment on seizure activity in FCD rats. We recorded ictal EEG activity in the prefrontal cortex during acute seizures induced by PTZ administration in FCD animals after treatment with Cef or PBS (Figure 7a). Status epilepticus was successfully recorded in the FCD animals after PTZ administration, as shown by the representative EEG traces and corresponding time‐frequency analysis (Figure 7b,c). At 2 h post‐PTZ seizure induction, PBS‐treated FCD rats exhibited 66.7% survival and 33.3% mortality rates, while Cef‐treated FCD rats showed 80% survival and 20% mortality. Although Cef treatment demonstrated an increase in survival and reduction in mortality rates following PTZ‐induced seizures, these differences were statistically nonsignificant (Figure 7d). Cef‐treated FCD rats exhibited prolonged latency to PTZ‐induced seizures compared to PBS‐treated controls, though this difference lacked statistical significance (Figure 7e). Notably, Cef treatment significantly reduced both seizure frequency and seizure duration within 2 h post‐PTZ administration (Figure 7f,g, *p < 0.05, ***p < 0.001, *n* = 8 for each group). These findings demonstrate that pharmacological enhancement of GLT‐1 with Cef attenuates epileptiform activity in FCD models.

### 3.8 Effects of α‐Syn on Seizure Activity Induced by PTZ in FCD Animals

**FIGURE 8:**
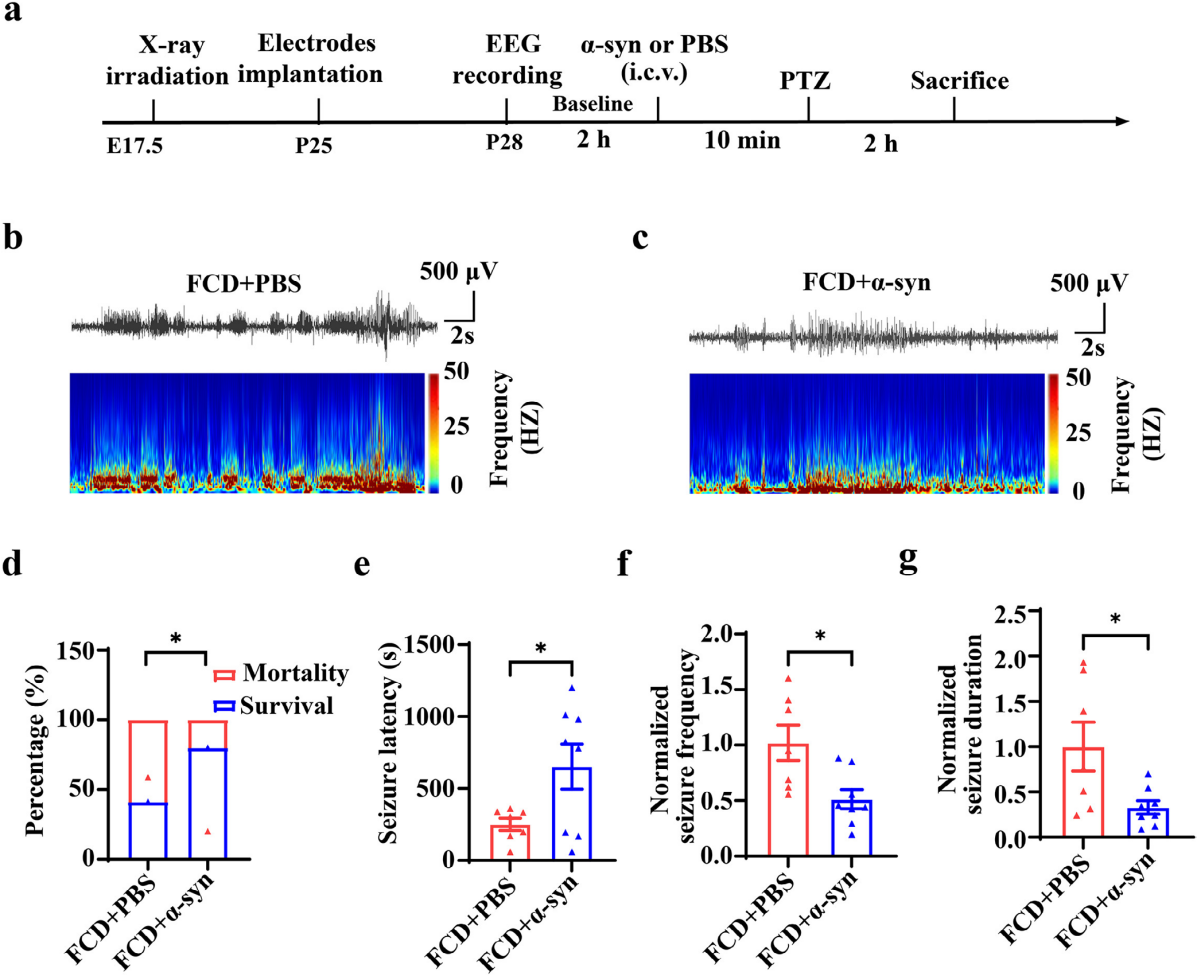
α‐syn exerted anti‐seizure effects in FCD animals. (a) Experimental paradigm. (b, c) Representative EEGs and corresponding time‐frequency plots recorded at the frontal cortex during the acute seizure discharges in FCD rats pretreated with PBS or α‐syn via lateral ventricle injection. (d) α‐syn significantly increased the survival rate of FCD animals after PTZ injection (*p < 0.05, two‐tailed chi‐square test, *n* = 10–17 for each group). (e) α‐syn significantly increased the latency of acute seizures induced by PTZ in FCD animals (*p < 0.05, unpaired two‐tailed *t*‐test, *n* = 7–8 for each group). (f, g) Statistics showing that α‐syn pretreatment significantly reduced both the seizure frequency and duration induced by PTZ in FCD animals (*p < 0.05, unpaired two‐tailed *t*‐test, *n* = 7–8 for each group).

Finally, we assessed α‐syn (300 μM, 250 μL, i.c.v.) effects on PTZ‐induced seizures in FCD rats using modified methods [43] (Figure 8a). Continuous EEG monitoring successfully recorded seizure activities in the frontal cortex of FCD rats (Figure 8b,c). Notably, α‐syn significantly reduced the mortality rate from 58.8% to 20% in FCD animals after PTZ‐induction (Figure 8d, *p < 0.05, *n* = 10–17 for each group). Seizure latency was significantly prolonged by α‐syn administration (Figure 8e, *p < 0.05, *n* = 7–8 for each group). Additionally, α‐syn efficiently reduced the seizure frequency and duration versus PBS controls in FCD rats (Figure 8f,g, *p < 0.05, *n* = 7–8 for each group). These results demonstrate α‐syn attenuates epileptiform activity in FCD models.

## 4 Discussion

Here, we identified significantly reduced mTOR/p‐mTOR ratio alongside substantially decreased GLT‐1 expression in cortical lesions of patients with FCD IIb and TSC. Critically, mTOR expression showed a significant negative correlation with α‐syn and GLT‐1 levels, whereas GLT‐1 expression exhibited a positive correlation with α‐syn levels in FCD lesions. Following mTOR inhibition or GLT‐1 up‐regulation, pathological p‐α‐syn accumulation was reversed, and seizure activities were effectively alleviated, suggesting the potential role of mTOR/GLT‐1 axis in the development of α‐syn pathology in FCD IIb and TSC.

Studies have shown that abnormal mTOR pathway activation exists in FCD and TSC lesions [54]. Here in the study, we identified intense expression of mTOR and p‐mTOR in the dysmorphic cells, including DNs, BCs, and GCs in the cortical lesions of patients with FCD IIb and TSC, and the mTOR/p‐mTOR ratio was correlated with the expression of α‐syn, further indicating the aberrant mTOR signaling pathway in the dysmorphic cells. Electrophysiological recording has shown that the dysmorphic cytomegalic neurons showed signs of hyperexcitability, with a very high membrane capacity and extremely low input resistance, attributing to the generation of epileptic activity in pediatric cortical dysplasia [55, 56]. Whereas BCs and GCs, large cells with atypical morphology, did not display active voltage‐ or ligand‐gated currents and did not appear to receive synaptic inputs [57]. α‐syn is a critical mediator in regulating synaptic transmission and maintaining synaptic plasticity, and extracellular α‐syn levels are regulated by neuronal activity [58]. In our previous studies, we have found that α‐syn was intensely expressed in DNs of FCD IIb lesions, while not expressed in BCs and GCs [12]. Thus, the activation of mTOR signaling is very likely to contribute to the aberrant expression of α‐syn in DNs, thereby mediating their hyperexcitability.

GLT‐1 is one of the major transporters that take up synaptic glutamate to maintain its optimal extracellular levels, thus preventing its accumulation in the synaptic cleft and the ensuing excitotoxicity [59]. Previous studies have shown that the expression of GLT‐1 is reduced, and inhibition of GLT‐1 decreases the threshold for evoking epileptiform activity in the freeze‐induced model of cortical dysplasia [60]. Here, we found that GLT‐1 expression is reduced in the cortical lesions of patients with FCD IIb and TSC, and FCD model with X‐ray irradiation. Notably, we found GLT‐1 expression level is correlated with the mTOR/p‐mTOR ratio in FCD lesions, and inhibition of mTOR signaling with rapamycin restored the expression of GLT‐1, further suggesting the causal role of mTOR signaling in regulating GLT‐1 function in FCD lesions. Additionally, we observed significant GLT‐1 expression in the cytoplasm of astrocytes in cortical lesions of patients with FCD IIb and TSC, as well as in the FCD model, highly likely contributing to the astrocytic production and recycle of glutamate in FCD lesions as well.

We observed elevated mTOR expression and reduced GLT‐1 levels in FCD lesions, consistent with findings in human FCD I and II specimens [15]. As a master regulator of cellular growth, mTOR modulates GLT‐1 via the mTOR–Akt–NF‐κB pathway in various neurological disorders [61, 62, 63], and astrocyte‐specific mTOR deletion alters GLT‐1 stability, glutamate clearance, and seizure susceptibility in animals [64]. Additionally, GLT‐1‐mediated glutamate excitotoxicity is linked to pathological p‐α‐syn deposition in synucleinopathy and Parkinson's disease models [21, 65]. In our FCD models, GLT‐1 potentiation via Cef rescued α‐syn depletion and pathological p‐α‐syn in FCD lesions and ameliorated PTZ‐induced acute seizure. These results confirm a critical modulatory role of GLT‐1 in the FCD‐associated seizures via regulating pathological p‐α‐syn. However, further investigation is needed to delineate the precise mechanism through which mTOR, GLT‐1, and α‐synuclein interact to drive FCD pathology. Moreover, intracerebroventricular α‐syn administration attenuated PTZ‐induced seizures in FCD models, likely mediated through enhanced synaptic glutamate clearance and α‐syn‐dependent interaction with presynaptic NMDA receptor activity [12].

In summary, our results establish the mTOR/GLT‐1 signaling axis as a key regulator of α‐syn pathology in FCD IIb and TSC, advancing mechanistic understanding of epileptogenesis in mTORopathies.

## 5 Limitations

Human FCD IIb and TSC are genetic disorders, primarily caused by somatic mutations in the mTOR pathway genes (e.g., TSC1, TSC2, DEPDC5, MTOR itself) [66, 67], which result in constitutive mTOR hyperactivation, driving all downstream pathological cellular events [68]. Genetic FCD IIb and TSC are often characterized by discrete, focal lesions within an otherwise normal‐appearing cortex [5, 69]. The X‐ray irradiation model, depending on the technique, often creates a more diffuse or widespread cortical abnormality [70]. This limits the study of the intricate interactions between the dysplastic focus and the “normal” surrounding cortex, which is crucial for understanding epileptogenesis. Our model, in contrast, uses an external physical insult (X‐ray irradiation) to disrupt cortical development. While we demonstrate that this insult also leads to mTOR activation, it is a secondary, downstream consequence of cellular injury and reactive processes. Therefore, our model creates a phenocopy (a similar appearance) rather than a true genocopy (a similar genetic cause) of the human conditions. Notwithstanding these limitations, the X‐ray‐induced FCD model remains a valuable and accessible tool [70, 71].

## 6 Conclusions

This study demonstrates that reduced significantly mTOR/p‐mTOR ratio and GLT‐1, along with markedly decreased α‐syn expression in cortical lesions of patients with FCD IIb and TSC. mTOR expression was negatively correlated with both α‐syn and GLT‐1, whereas GLT‐1 expression was positively correlated with α‐syn. Importantly, mTOR inhibition or GLT‐1 up‐regulation reversed pathological p‐α‐syn accumulation and effectively alleviated seizure activity, indicating that the mTOR/GLT‐1 axis may drive α‐syn pathology in FCD IIb and TSC.

## Author Contributions

**Kai‐Feng Shen**, **Chun‐Qing Zhang**, **Hui Yang**, and **Ping Liang:** designed the study and revised the manuscript. **Li Zhang** and **Jian‐Ping Song:** performed the experiments, analyzed the data, and wrote the manuscript. **Jun Huang:** collected the clinical samples.

## Funding

This work was supported by Young and Middle‐aged Medical Talents of Chongqing (2024GDRC013), the Natural Science Foundation of Chongqing (CSTB2023NSCQ‐MSX0216), and Personnel Development Project of Xinqiao Hospital (2024XKRC007).

## Ethics Statement

The clinical samples used in this study were obtained in compliance with the principles of the Declaration of Helsinki and were approved by the Ethics Committee of Xinqiao Hospital, Army Medical University (Approval No. 2022‐361‐01). All the animal experimental procedures were reviewed and approved by the Internal Animal Care and Use Committee of the Army Medical University (AMUWEC20226244).

## Consent

This research involved human subjects, and the patient consent statement is applicable for this research. All procedures performed in studies involving animals were in accordance with the ethical standards of the institution or practice at which the studies were conducted.

## Conflicts of Interest

The authors declare no conflicts of interest.

## Supporting information


**Figure S1:** Ceftriaxone treatment effectively enhanced the expression of GLT‐1 mRNA and protein. (a) RT‐PCR results showing that the reduced expression of Glt‐1 mRNA in FCD animals were rescued by Cef treatment (*p < 0.05, ***p < 0.001, one‐way ANOVA test, *n* = 6 for each group). (b, c) Representative images showing weakened GLT‐1 immunoreactivity was restored by Cef in FCD rats (***p < 0.001, one‐way ANOVA test, *n* = 7 for each group). Arrow indicated the expression of GLT‐1 in the cytoplasm of astrocytes. Scale bars: 50 μm for upper panel, 25 μm for bottom panel.

**Table S1:** PCR primers used in this study.
**Table S2:** The detailed information of antibodies for western blotting and immunostaing.

## Data Availability

The data that support the findings of this study are available from the corresponding author upon reasonable request.

## References

[1] J. Banerjee , S. Dey , A. B. Dixit , et al., “GABAA Receptor‐Mediated Epileptogenicity in Focal Cortical Dysplasia (FCD) Depends on Age at Epilepsy Onset,” Frontiers in Cellular Neuroscience 14 (2020): 562811, 10.3389/fncel.2020.562811. 33192309 PMC7556289

[2] D. M. Talos , H. Sun , B. Kosaras , et al., “Altered Inhibition in Tuberous Sclerosis and Type IIb Cortical Dysplasia,” Annals of Neurology 71 (2012): 539–551, 10.1002/ana.22696. 22447678 PMC3334406

[3] S. Adler , S. Lorio , T. S. Jacques , et al., “Towards In Vivo Focal Cortical Dysplasia Phenotyping Using Quantitative MRI,” NeuroImage: Clinical 15 (2017): 95–105, 10.1016/j.nicl.2017.04.017. 28491496 PMC5413300

[4] J. M. Peters , R. R. Struyven , A. K. Prohl , et al., “White Matter Mean Diffusivity Correlates With Myelination in Tuberous Sclerosis Complex,” Annals of Clinical Translational Neurology 6 (2019): 1178–1190, 10.1002/acn3.793. 31353853 PMC6649396

[5] A. Arena , T. S. Zimmer , J. van Scheppingen , et al., “Oxidative Stress and Inflammation in a Spectrum of Epileptogenic Cortical Malformations: Molecular Insights Into Their Interdependence,” Brain Pathology 29 (2019): 351–365, 10.1111/bpa.12661. 30303592 PMC8028690

[6] C.‐Q. Zhang , H.‐F. Shu , Q. Yin , et al., “Expression and Cellular Distribution of Vascular Endothelial Growth Factor‐C System in Cortical Tubers of the Tuberous Sclerosis Complex,” Brain Pathology 22 (2012): 205–218, 10.1111/j.1750-3639.2011.00519.x. 21767323 PMC8029110

[7] F.‐J. Sun , C.‐Q. Zhang , X. Chen , et al., “Downregulation of CD47 and CD200 in Patients With Focal Cortical Dysplasia Type IIb and Tuberous Sclerosis Complex,” Journal of Neuroinflammation 13 (2016): 85, 10.1186/s12974-016-0546-2. 27095555 PMC4837553

[8] T. Scholl , V.‐E. Gruber , S. Samueli , et al., “Neurite Outgrowth Inhibitor (NogoA) is Upregulated in White Matter Lesions of Complex Cortical Malformations,” Journal of Neuropathology and Experimental Neurology 80 (2021): 274–282, 10.1093/jnen/nlaa159. 33517425

[9] M. Bozic , M. Caus , R. R. Rodrigues‐Diez , et al., “Protective Role of Renal Proximal Tubular Alpha‐Synuclein in the Pathogenesis of Kidney Fibrosis,” Nature Communications 11 (2020): 1943, 10.1038/s41467-020-15732-9. PMC7181766 32327648

[10] J. Zhang , T. Cai , F. Zhao , et al., “The Role of α‐Synuclein and Tau Hyperphosphorylation‐Mediated Autophagy and Apoptosis in Lead‐Induced Learning and Memory Injury,” International Journal of Biological Sciences 8 (2012): 935–944, 10.7150/ijbs.4499. 22811615 PMC3399316

[11] S. Elfarrash , N. M. Jensen , N. Ferreira , et al., “Organotypic Slice Culture Model Demonstrates Inter‐Neuronal Spreading of Alpha‐Synuclein Aggregates,” Acta Neuropathologica Communications 7 (2019): 213, 10.1186/s40478-019-0865-5. 31856920 PMC6924077

[12] L. Zhang , J. Huang , L. Dai , et al., “Expression Profiles of α‐Synuclein in Cortical Lesions of Patients With FCD IIb and TSC, and FCD Rats,” Frontiers in Neurology 14 (2023): 1255097, 10.3389/fneur.2023.1255097. 38020594 PMC10662349

[13] M. Laplante and D. M. Sabatini , “mTOR Signaling in Growth Control and Disease,” Cell 149 (2012): 274–293, 10.1016/j.cell.2012.03.017. 22500797 PMC3331679

[14] S. J. Tang , G. Reis , H. Kang , A. C. Gingras , N. Sonenberg , and E. M. Schuman , “A Rapamycin‐Sensitive Signaling Pathway Contributes to Long‐Term Synaptic Plasticity in the Hippocampus,” Proceedings of the National Academy of Sciences of the United States of America 99 (2002): 467–472, 10.1073/pnas.012605299. 11756682 PMC117583

[15] M. Guo , J. Zhang , J. Wang , et al., “Aberrant Adenosine Signaling in Patients With Focal Cortical Dysplasia,” Molecular Neurobiology 60 (2023): 4396–4417, 10.1007/s12035-023-03351-6. 37103687 PMC10330374

[16] M. R. Khan , X. Yin , S.‐U. Kang , et al., “Enhanced mTORC1 Signaling and Protein Synthesis in Pathologic α‐Synuclein Cellular and Animal Models of Parkinson's Disease,” Science Translational Medicine 15 (2023): eadd0499, 10.1126/scitranslmed.add0499. 38019930

[17] B. I. Pérez‐Revuelta , M. M. Hettich , A. Ciociaro , et al., “Metformin Lowers Ser‐129 Phosphorylated α‐Synuclein Levels via mTOR‐Dependent Protein Phosphatase 2A Activation,” Cell Death & Disease 5 (2014): e1209, 10.1038/cddis.2014.175. 24810045 PMC4047877

[18] L. F. McNair , J. V. Andersen , B. I. Aldana , et al., “Deletion of Neuronal GLT‐1 in Mice Reveals Its Role in Synaptic Glutamate Homeostasis and Mitochondrial Function,” Journal of Neuroscience 39 (2019): 4847–4863, 10.1523/JNEUROSCI.0894-18.2019. 30926746 PMC6670249

[19] C.‐S. Sung , Z.‐H. Wen , C.‐W. Feng , et al., “Potentiation of Spinal Glutamatergic Response in the Neuron‐Glia Interactions Underlies the Intrathecal IL‐1β‐Induced Thermal Hyperalgesia in Rats,” CNS Neuroscience & Therapeutics 23 (2017): 580–589, 10.1111/cns.12705. 28544775 PMC6492640

[20] M. Barker‐Haliski and H. S. White , “Glutamatergic Mechanisms Associated With Seizures and Epilepsy,” Cold Spring Harbor Perspectives in Medicine 5 (2015): a022863, 10.1101/cshperspect.a022863. 26101204 PMC4526718

[21] X. Wu , X. Meng , F. Tan , et al., “Regulatory Mechanism of miR‐543‐3p on GLT‐1 in a Mouse Model of Parkinson's Disease,” ACS Chemical Neuroscience 10 (2019): 1791–1800, 10.1021/acschemneuro.8b00683. 30676715

[22] Y. Zhang , X. Meng , Z. Jiao , Y. Liu , X. Zhang , and S. Qu , “Generation of a Novel Mouse Model of Parkinson's Disease via Targeted Knockdown of Glutamate Transporter GLT‐1 in the Substantia Nigra,” ACS Chemical Neuroscience 11 (2020): 406–417, 10.1021/acschemneuro.9b00609. 31909584

[23] K. H. Jeong , K.‐O. Cho , M.‐Y. Lee , S. Y. Kim , and W. J. Kim , “Vascular Endothelial Growth Factor Receptor‐3 Regulates Astroglial Glutamate Transporter‐1 Expression via mTOR Activation in Reactive Astrocytes Following Pilocarpine‐Induced Status Epilepticus,” Glia 69 (2021): 296–309, 10.1002/glia.23897. 32835451

[24] I. Blumcke , F. Cendes , H. Miyata , M. Thom , E. Aronica , and I. Najm , “Toward a Refined Genotype–Phenotype Classification Scheme for the International Consensus Classification of Focal Cortical Dysplasia,” Brain Pathology 31 (2021): e12956, 10.1111/bpa.12956. 34196989 PMC8412090

[25] V. Gruber , M. J. Luinenburg , K. Colleselli , et al., “Increased Expression of Complement Components in Tuberous Sclerosis Complex and Focal Cortical Dysplasia Type 2B Brain Lesions,” Epilepsia 63 (2022): 364–374, 10.1111/epi.17139. 34904712 PMC9299842

[26] M. R. Hynd , J. M. Lewohl , H. L. Scott , and P. R. Dodd , “Biochemical and Molecular Studies Using Human Autopsy Brain Tissue,” Journal of Neurochemistry 85 (2003): 543–562, 10.1046/j.1471-4159.2003.01747.x. 12694381

[27] Y. Liu , X. Zhang , W. Lin , N. Kehriman , W. Kuang , and X. Ling , “Multi‐Factor Combined Biomarker Screening Strategy to Rapidly Diagnose Alzheimer's Disease and Evaluate Drug Effect Based on a Rat Model,” Journal of Pharmaceutical Analysis 12 (2022): 627–636, 10.1016/j.jpha.2022.04.003. 36105160 PMC9463486

[28] C. Kellinghaus , T. Kunieda , Z. Ying , A. Pan , H. O. Lüders , and I. M. Najm , “Severity of Histopathologic Abnormalities and In Vivo Epileptogenicity in the in Utero Radiation Model of Rats Is Dose Dependent,” Epilepsia 45 (2004): 583–591, 10.1111/j.0013-9580.2004.41103.x. 15144422

[29] T. Niedermair , C. Lukas , S. Li , et al., “Influence of Extracellular Vesicles Isolated From Osteoblasts of Patients With Cox‐Arthrosis and/or Osteoporosis on Metabolism and Osteogenic Differentiation of BMSCs,” Frontiers in Bioengineering and Biotechnology 8 (2020): 615520, 10.3389/fbioe.2020.615520. 33425878 PMC7785908

[30] Z. Wang , R. Xie , X. Yang , et al., “Female Mice Lacking ERβ Display Excitatory/Inhibitory Synaptic Imbalance to Drive the Pathogenesis of Temporal Lobe Epilepsy,” Theranostics 11 (2021): 6074–6089, 10.7150/thno.56331. 33897900 PMC8058727

[31] F. Wang , Y.‐J. Yang , N. Yang , et al., “Enhancing Oligodendrocyte Myelination Rescues Synaptic Loss and Improves Functional Recovery After Chronic Hypoxia,” Neuron 99 (2018): 689–701.e5, 10.1016/j.neuron.2018.07.017. 30078577 PMC6170028

[32] I. Paakkari , “A Simple Method for the Verification of a Successful Cannulation of the Rat Cerebral Ventricles,” Experientia 36 (1980): 887–889, 10.1007/BF01978633. 7398862

[33] X. Wang , S. Kimura , T. Yazawa , and N. Endo , “Cerebrospinal Fluid Sampling by Lumbar Puncture in Rats—Repeated Measurements of Nitric Oxide Metabolites,” Journal of Neuroscience Methods 145 (2005): 89–95, 10.1016/j.jneumeth.2004.12.002. 15922028

[34] Z. Liang , Y. Miao , X. Teng , et al., “Hydrogen Sulfide Inhibits Ferroptosis in Cardiomyocytes to Protect Cardiac Function in Aging Rats,” Frontiers in Molecular Biosciences 9 (2022): 947778, 10.3389/fmolb.2022.947778. 35936785 PMC9355033

[35] C. Ghosh , R. Myers , C. O'Connor , et al., “Cortical Dysplasia in Rats Provokes Neurovascular Alterations, GLUT1 Dysfunction and Metabolic Disturbances That Are Sustained Post‐Seizure Induction,” Molecular Neurobiology 59 (2022): 2389–2406, 10.1007/s12035-021-02624-2. 35084654 PMC9018620

[36] A. D. Nemes , R. O'Dwyer , I. M. Najm , Z. Ying , J. Gonzalez‐Martinez , and A. V. Alexopoulos , “Treatment With Lacosamide Impedes Generalized Seizures in a Rodent Model of Cortical Dysplasia,” Epilepsia 58 (2017): 1755–1761, 10.1111/epi.13856. 28833036

[37] J. Tchekalarova , H. Kubová , and P. Mareš , “Postnatal Caffeine Treatment Affects Differently Two Pentylenetetrazol Seizure Models in Rats,” Seizure 18 (2009): 463–469, 10.1016/j.seizure.2009.04.002. 19493686

[38] A.‐M. Costa , L. Senn , L. Anceschi , V. Brighenti , F. Pellati , and G. Biagini , “Antiseizure Effects of Fully Characterized Non‐Psychoactive *cannabis sativa* L. Extracts in the Repeated 6‐Hz Corneal Stimulation Test,” Pharmaceuticals 14 (2021): 1259, 10.3390/ph14121259. 34959660 PMC8703309

[39] S. Tang , T. Wang , X. Zhang , et al., “Olfactomedin‐3 Enhances Seizure Activity by Interacting With AMPA Receptors in Epilepsy Models,” Frontiers in Cell and Development Biology 8 (2020): 722, 10.3389/fcell.2020.00722. PMC7431667 32850838

[40] N. Z. Gcwensa , D. L. Russell , K. Y. Long , et al., “Excitatory Synaptic Structural Abnormalities Produced by Templated Aggregation of α‐Syn in the Basolateral Amygdala,” Neurobiology of Disease 199 (2024): 106595, 10.1016/j.nbd.2024.106595. 38972360 PMC11632701

[41] E. K. Erickson , A. J. DaCosta , S. C. Mason , et al., “Cortical Astrocytes Regulate Ethanol Consumption and Intoxication in Mice,” Neuropsychopharmacology 46 (2021): 500–508, 10.1038/s41386-020-0721-0. 32464636 PMC8027025

[42] M. A. Khanfar , S. K. Bardaweel , M. R. Akl , and K. A. El Sayed , “Olive Oil‐Derived Oleocanthal as Potent Inhibitor of Mammalian Target of Rapamycin: Biological Evaluation and Molecular Modeling Studies,” Phytotherapy Research 29 (2015): 1776–1782, 10.1002/ptr.5434. 26248874 PMC5051273

[43] C. M. Drion , J. van Scheppingen , A. Arena , et al., “Effects of Rapamycin and Curcumin on Inflammation and Oxidative Stress In Vitro and In Vivo—In Search of Potential Anti‐Epileptogenic Strategies for Temporal Lobe Epilepsy,” Journal of Neuroinflammation 15 (2018): 212, 10.1186/s12974-018-1247-9. 30037344 PMC6056921

[44] H. A. Lashuel , C. R. Overk , A. Oueslati , and E. Masliah , “The Many Faces of α‐Synuclein: From Structure and Toxicity to Therapeutic Target,” Nature Reviews. Neuroscience 14 (2013): 38–48, 10.1038/nrn3406. 23254192 PMC4295774

[45] K. Wu , J. Yue , K. Shen , et al., “Increased Expression of Fibroblast Growth Factor 13 in Cortical Lesions of the Focal Cortical Dysplasia,” Brain Research Bulletin 168 (2021): 36–44, 10.1016/j.brainresbull.2020.11.023. 33285262

[46] J. D. Rothstein , S. Patel , M. R. Regan , et al., “Beta‐Lactam Antibiotics Offer Neuroprotection by Increasing Glutamate Transporter Expression,” Nature 433 (2005): 73–77, 10.1038/nature03180. 15635412

[47] C. Thöne‐Reineke , C. Neumann , P. Namsolleck , et al., “The Beta‐Lactam Antibiotic, Ceftriaxone, Dramatically Improves Survival, Increases Glutamate Uptake and Induces Neurotrophins in Stroke,” Journal of Hypertension 26 (2008): 2426–2435, 10.1097/HJH.0b013e328313e403. 19008722

[48] Y. Zhang , X. Zhang , and S. Qu , “Ceftriaxone Protects Astrocytes From MPP(+) via Suppression of NF‐κB/JNK/c‐Jun Signaling,” Molecular Neurobiology 52 (2015): 78–92, 10.1007/s12035-014-8845-z. 25112679

[49] T. C. H. Leung , C. N. P. Lui , L. W. Chen , W. H. Yung , Y. S. Chan , and K. K. L. Yung , “Ceftriaxone Ameliorates Motor Deficits and Protects Dopaminergic Neurons in 6‐Hydroxydopamine‐Lesioned Rats,” ACS Chemical Neuroscience 3 (2012): 22–30, 10.1021/cn200072h. 22860178 PMC3369786

[50] O. Gunduz , C. Oltulu , and A. Ulugol , “Role of GLT‐1 Transporter Activation in Prevention of Cannabinoid Tolerance by the β‐Lactam Antibiotic, Ceftriaxone, in Mice,” Pharmacology, Biochemistry, and Behavior 99 (2011): 100–103, 10.1016/j.pbb.2011.04.012. 21536061

[51] J. K. Hefendehl , J. LeDue , R. W. Y. Ko , J. Mahler , T. H. Murphy , and B. A. MacVicar , “Mapping Synaptic Glutamate Transporter Dysfunction In Vivo to Regions Surrounding aβ Plaques by iGluSnFR Two‐Photon Imaging,” Nature Communications 7 (2016): 13441, 10.1038/ncomms13441. PMC5114608 27834383

[52] O. A. Abulseoud , J. D. Miller , J. Wu , D. S. Choi , and D. P. Holschneider , “Ceftriaxone Upregulates the Glutamate Transporter in Medial Prefrontal Cortex and Blocks Reinstatement of Methamphetamine Seeking in a Condition Place Preference Paradigm,” Brain Research 1456 (2012): 14–21, 10.1016/j.brainres.2012.03.045. 22521042 PMC3922613

[53] S. K. Hota , K. Barhwal , K. Ray , S. B. Singh , and G. Ilavazhagan , “Ceftriaxone Rescues Hippocampal Neurons From Excitotoxicity and Enhances Memory Retrieval in Chronic Hypobaric Hypoxia,” Neurobiology of Learning and Memory 89 (2008): 522–532, 10.1016/j.nlm.2008.01.003. 18304843

[54] H. Miyata , A. C. Y. Chiang , and H. V. Vinters , “Insulin Signaling Pathways in Cortical Dysplasia and TSC‐Tubers: Tissue Microarray Analysis,” Annals of Neurology 56 (2004): 510–519, 10.1002/ana.20234. 15455398

[55] C. Cepeda , R. S. Hurst , J. Flores‐Hernández , et al., “Morphological and Electrophysiological Characterization of Abnormal Cell Types in Pediatric Cortical Dysplasia,” Journal of Neuroscience Research 72 (2003): 472–486, 10.1002/jnr.10604. 12704809

[56] S. Abdijadid , G. W. Mathern , M. S. Levine , and C. Cepeda , “Basic Mechanisms of Epileptogenesis in Pediatric Cortical Dysplasia,” CNS Neuroscience & Therapeutics 21 (2015): 92–103, 10.1111/cns.12345. 25404064 PMC4442638

[57] J. Zhang , D. Argueta , X. Tong , H. V. Vinters , G. W. Mathern , and C. Cepeda , “Iconography of Abnormal Non‐Neuronal Cells in Pediatric Focal Cortical Dysplasia Type IIb and Tuberous Sclerosis Complex,” Frontiers in Cellular Neuroscience 18 (2025): 1486315, 10.3389/fncel.2024.1486315. 39835291 PMC11743721

[58] K. Yamada and T. Iwatsubo , “Extracellular α‐Synuclein Levels Are Regulated by Neuronal Activity,” Molecular Neurodegeneration 13 (2018): 9, 10.1186/s13024-018-0241-0. 29467003 PMC5822605

[59] E. Pajarillo , A. Rizor , J. Lee , M. Aschner , and E. Lee , “The Role of Astrocytic Glutamate Transporters GLT‐1 and GLAST in Neurological Disorders: Potential Targets for Neurotherapeutics,” Neuropharmacology 161 (2019): 107559, 10.1016/j.neuropharm.2019.03.002. 30851309 PMC6731169

[60] S. L. Campbell and J. J. Hablitz , “Decreased Glutamate Transport Enhances Excitability in a Rat Model of Cortical Dysplasia,” Neurobiology of Disease 32 (2008): 254–261, 10.1016/j.nbd.2008.07.003. 18674619 PMC2643870

[61] Y.‐F. Ji , L. Zhou , Y.‐J. Xie , et al., “Upregulation of Glutamate Transporter GLT‐1 by mTOR‐Akt‐NF‐кB Cascade in Astrocytic Oxygen‐Glucose Deprivation,” Glia 61 (2013): 1959–1975, 10.1002/glia.22566. 24108520

[62] W.‐Y. Huang , C. Jiang , H.‐B. Ye , et al., “miR‐124 Upregulates Astrocytic Glutamate Transporter‐1 via the Akt and mTOR Signaling Pathway Post Ischemic Stroke,” Brain Research Bulletin 149 (2019): 231–239, 10.1016/j.brainresbull.2019.04.013. 31004734

[63] A. Alotaibi , K. K. Bhowmik , W. Wong , et al., “Modulatory Effects of GLT‐1 Enhancer, MC‐100093, on Glutamate Uptake and Associated Signaling Pathways in Female and Male Alcohol Preferring Rats Exposed to Ethanol,” International Journal of Neuropsychopharmacology 28 (2025): pyaf075, 10.1093/ijnp/pyaf075. 41055573 PMC12581845

[64] X. Wang , L. Sha , N. Sun , Y. Shen , and Q. Xu , “Deletion of mTOR in Reactive Astrocytes Suppresses Chronic Seizures in a Mouse Model of Temporal Lobe Epilepsy,” Molecular Neurobiology 54 (2017): 175–187, 10.1007/s12035-015-9590-7. 26732600

[65] L. P. Diniz , A. P. B. Araujo , I. Matias , et al., “Astrocyte Glutamate Transporters Are Increased in an Early Sporadic Model of Synucleinopathy,” Neurochemistry International 138 (2020): 104758, 10.1016/j.neuint.2020.104758. 32439533

[66] T. S. Zimmer , A. Korotkov , S. Zwakenberg , et al., “Upregulation of the Pathogenic Transcription Factor SPI1/PU.1 in Tuberous Sclerosis Complex and Focal Cortical Dysplasia by Oxidative Stress,” Brain Pathology 31 (2021): e12949, 10.1111/bpa.12949. 33786950 PMC8412124

[67] D. García‐Rincón , J. Díaz‐Alonso , J. Paraíso‐Luna , et al., “Contribution of Altered Endocannabinoid System to Overactive mTORC1 Signaling in Focal Cortical Dysplasia,” Frontiers in Pharmacology 9 (2019): 1508, 10.3389/fphar.2018.01508. 30687088 PMC6334222

[68] J. van Scheppingen , D. W. M. Broekaart , T. Scholl , et al., “Dysregulation of the (Immuno)proteasome Pathway in Malformations of Cortical Development,” Journal of Neuroinflammation 13 (2016): 202, 10.1186/s12974-016-0662-z. 27566410 PMC5002182

[69] D. W. M. Broekaart , J. van Scheppingen , J. J. Anink , et al., “Increased Matrix Metalloproteinases Expression in Tuberous Sclerosis Complex: Modulation by microRNA 146a and 147b In Vitro,” Neuropathology and Applied Neurobiology 46 (2020): 142–159, 10.1111/nan.12572. 31183875 PMC7217197

[70] G. Battaglia , A. J. Becker , J. LoTurco , et al., “Basic Mechanisms of MCD in Animal Models,” Epileptic Disorders: International Epilepsy Journal with Videotape 11 (2009): 206–214, 10.1684/epd.2009.0273. 19740719 PMC4362697

[71] M. Wong , “Animal Models of Focal Cortical Dysplasia and Tuberous Sclerosis Complex: Recent Progress Toward Clinical Applications,” Epilepsia 50 (2009): 34–44, 10.1111/j.1528-1167.2009.02295.x. PMC3934654 19761452

