# Supplementary figures and images for: The Mechanism Underlying the Abnormal Expression of α‐Synuclein in the Cortical Lesions of Patients With FCD Type IIb and TSC
Source: CNS Neurosci Ther. 2026 Apr 24;32(4):e70893. doi: 10.1002/cns.70893 (PMC13108419; doi:10.1002/cns.70893)

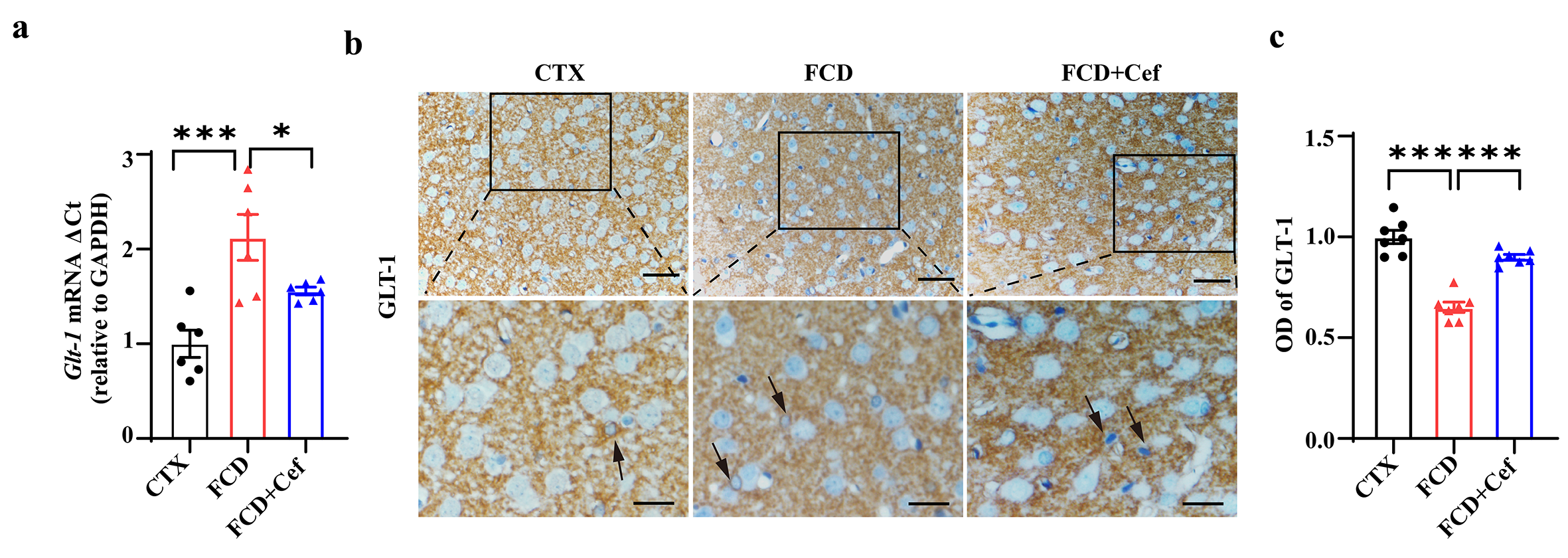

Supplement: Supplementary file 1 — Figure S1: Ceftriaxone treatment effectively enhanced the expression of GLT‐1 mRNA and protein. (a) RT‐PCR results showing that the reduced expression of Glt‐1 mRNA in FCD animals were rescued by Cef treatment (*p < 0.05, ***p < 0.001, one‐way ANOVA test, n = 6 for each group). (b, c) Representative images showing weakened GLT‐1 immunoreactivity was restored by Cef in FCD rats (***p < 0.001, one‐way ANOVA test, n = 7 for each group). Arrow indicated the expression of GLT‐1 in the cytoplasm of astrocytes. Scale bars: 50 μm for upper panel, 25 μm for bottom panel. [file CNS-32-e70893-s001.tif]
