# Supplementary material for: The Mechanism Underlying the Abnormal Expression of α‐Synuclein in the Cortical Lesions of Patients With FCD Type IIb and TSC
Source: CNS Neurosci Ther. 2026 Apr 24;32(4):e70893. doi: 10.1002/cns.70893 (PMC13108419; doi:10.1002/cns.70893)
Supplement: Supplementary file 2 — Table S1: PCR primers used in this study. Table S2: The detailed information of antibodies for western blotting and immunostaing. [file CNS-32-e70893-s002.docx]

Table S1. PCR primers used in this study.

|  | Forward | Reverse | Exon location |
| --- | --- | --- | --- |
| ***mTOR****-rat* | 5'CTCGGCACATCACTCCCTTC3' | 5'GCAACAACGGCTTTCCACC3' | exon 7 |
| ***α-syn-****rat* | 5'GTTTTGTCAAGAAGGACCAGATG3' | 5'GGCATTTCATAAGCCTCACTG3' | exon 4–5 junction |
| ***Glt-1****-rat* | 5'TGTCTATGCCGCACACAACT3' | 5'GGTCTCGATATCCAGGAATGGG3' | exon 10 |
| ***Gapdh****-rat* | 5'AAGTTCAACGGCACAGTCAAGG3' | 5'CGCCAGTAGACTCCACGACATA3' | exon 3–4 junction |

Table S2. The detailed information of antibodies for western blotting and immunostaing.

| **Antibody** | **Manufacturer and number** | **Dilution** |
| --- | --- | --- |
| mTOR | cat.no:28273-1-AP，Proteintech | 1:100 |
| p-mTOR (Ser2448) | cat.no: 67778-1-IG，Proteintech | 1:500 |
| mTOR | cat.no:2983T, CST | 1:1000 |
| p-mTOR (Ser2448) | cat.no:5536T,CST | 1:1000 |
| α-syn | cat.no:10842-1-AP,Proteintech | 1:200 |
| p-α-syn (Ser129) | cat.no:ab51253, abcam | 1:100 |
| GLT-1 | cat.no: 22515-1-AP, Proteintech | 1:500 |
| NeuN | cat.no:ab104224,Abcam | 1:5000 |
| NeuN | cat.no:OB-PRBO39, Oasis Biofarm | 1:500 |
| Vimentin | cat.no:660330-1-Ig, Proteintech | 1:500 |
| Vimentin | cat.no:D21H3, CST | 1:500 |
| VGLUT1 | cat.no:135304, Synaptic Systems | 1:500 |
| VAGT | cat.no:131011,Synaptic Systems | 1:500 |
| Tubulin | cat.no:66031-1-Ig,Proteintech | 1:20000 |
| HRP-conjugated goat anti-rabbit | cat.no:SA00001-2, Proteintech | 1:1000 |
| HRP-conjugated goat anti-mouse | cat.no: SA00001-1, Proteintech | 1:1000 |
| Alexa Fluor 488-conjugated mouse | cat.no:A-10680, Thermo Fisher Scientific | 1:1000 |
| Alexa Fluor 555-conjugated rabbit | cat.no:A-21428, Thermo Fisher Scientific | 1:1000 |
| Alexa Fluor 647-conjugated guinea pig | cat.no: A-21450, Thermo Fisher Scientific | 1:1000 |
| HRP-conjugated goat anti-rabbit | cat.no:A0352, Beyotime | 1:100 |
| HRP-conjugated goat anti-mouse | cat.no: A0350, Beyotime | 1:100 |
